# An amine protecting group deprotectable under nearly neutral oxidative conditions

**DOI:** 10.3762/bjoc.14.149

**Published:** 2018-07-13

**Authors:** Shahien Shahsavari, Chase McNamara, Mark Sylvester, Emily Bromley, Savannah Joslin, Bao-Yuan Lu, Shiyue Fang

**Affiliations:** 1 Department of Chemistry, Michigan Technological University, 1400 Townsend Drive, Houghton, MI 49931, USA; 2 Nalco Champion, an Ecolab Company, 11177 S. Stadium Drive, Sugar Land, TX 77478, USA

**Keywords:** amine, carbamate, dM-Dmoc, oxidation, protecting group

## Abstract

The 1,3-dithiane-based dM-Dmoc group was studied for the protection of amino groups. Protection was achieved under mild conditions for aliphatic amines, and under highly reactive conditions for the less reactive arylamines. Moderate to excellent yields were obtained. Deprotection was performed by oxidation followed by treating with a weak base. The yields were good to excellent. The new amino protecting group offers a different dimension of orthogonality in reference to the commonly used amino protecting groups in terms of deprotection conditions. It is expected to allow a collection of transformations to be carried out on the protected substrates that are unattainable using any known protecting groups.

## Introduction

**Scheme 1:**
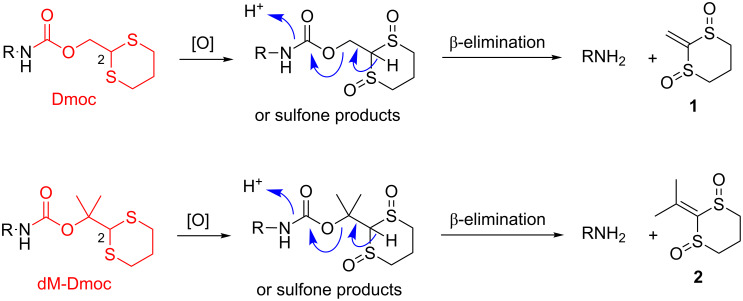
Dmoc and dM-Dmoc protection and deprotection of amines.

In multistep organic synthesis, amino groups usually have to be protected [1]. Protecting groups for the purpose mainly include those deprotectable by acid (e.g., *tert*-butyloxycarbonyl (Boc) group) [2–4], base (e.g., 9-fluorenylmethyloxycarbonyl (Fmoc) group and trifluoroacetyl group) [5–7], catalytic hydrogenation (e.g., benzyl group) [8], photoirradiation (e.g., 2-nitrophenylethyl carbamate and 6-nitroveratryl carbamate) [9–10] and fluoride (e.g., trimethylsilylethyloxycarbonyl (Teoc) group) [11–12]. The 1,3-dithian-2-ylmethoxycarbonyl (Dmoc) group first reported by Kunz and co-workers provides a different dimension of orthogonality of amine protection in terms of deprotection conditions [13–17]. This group was deprotected under oxidative conditions under which the commonly used Boc, Fmoc, benzyl and Teoc groups could potentially survive. Oxidation was achieved by hydrogen peroxide in the presence of an ammonium molybdate catalyst. Recently, we reported the use of the Dmoc group for amine protection in automated solid-phase oligodeoxynucleotide (ODN) synthesis [18]. For deprotection, we found that sodium periodate could effectively oxidize multiple Dmoc functions in the ODNs to achieve complete deprotection. Under these oxidative conditions, oxidation of the ODN was not observed. The mild deprotection conditions allowed us to introduce sensitive functionalities into ODNs, which are otherwise impossible or highly difficult to achieve [18]. In addition, we also investigated the potential of the dimethyl-1,3-dithian-2-ylmethyl (dM-Dim) group for orthogonal carboxylic acid protection [19]. To further explore the use of the 1,3-dithiane function as protecting group in organic synthesis, here we report the results of our studies on the use of the dimethyl-1,3-dithian-2-ylmethoxycarbonyl (dM-Dmoc) group for amine protection (Scheme 1). Compared with the Dmoc group, the dM-Dmoc group is expected to be more stable under nucleophilic conditions, which will allow many transformations including base hydrolysis of esters and amides, hydride reduction of carbonyl compounds, and a wide range of nucleophilic substitution reactions to be carried out without losing the protection. With Dmoc protection, such transformations would be unattainable or require fine tuning of reaction conditions to keep the protection. In addition, the side product **2** from deprotection of dM-Dmoc is less likely to act as a Michael acceptor to react with the amine product than **1** from deprotection of Dmoc due to its higher steric hindrance. Such side reactions could be a serious issue in some situations [18].

## Results and Discussion

To protect amines, compound **4** was prepared readily by reacting deprotonated 1,3-dithiane with acetone followed by treating with *p*-nitrophenylchloroformate (see experimental section). The compound is stable, which allows easy purification and storage. However, we expected that it could react with amines under suitable conditions. Using benzylamine (**3a**) as the model substrate, we tested a variety of reaction conditions to form the dM-Dmoc protected carbamate **5a** (see Table 1 for structures). These include using different solvents such as THF, DCM, acetonitrile and toluene, and different bases such as DIPEA, pyridine and trimethylamine. We found that the conditions most suitable for the reaction were to react one equivalent amine with one equivalent of **4** in the solvent THF using five equivalents of DIPEA as the base. At room temperature, the reaction could complete within eight hours.

**Table 1:** Protection of amines with dM-Dmoc and deprotection.^a^

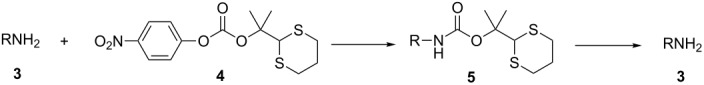			

entry	**3**	**5** (yield)	**3** (yield)

1	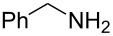 **3a**	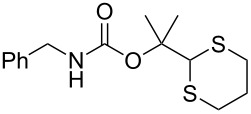 **5a** (92%)	**3a** (76%)
2	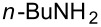 **3b**	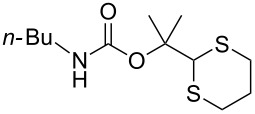 **5b** (89%)	**3b** (54%)
3	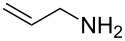 **3c**	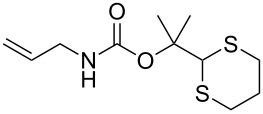 **5c** (72%)	**3c** (55%)
4	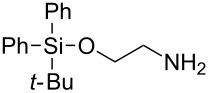 **3d**	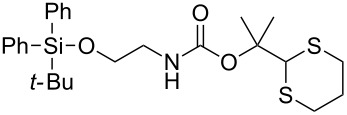 **5d** (97%)	**3d** (88%)
5	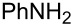 **3e**	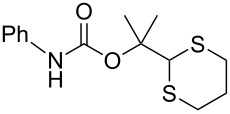 **5e** (46%)	**3e** (73%)
6	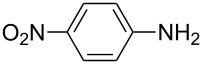 **3f**	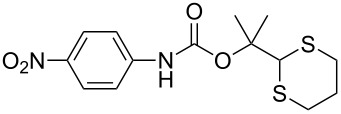 **5f** (42%)	**3f** (64%)
7	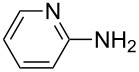 **3g**	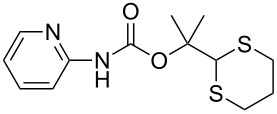 **5g** (57%)	**3g** (53%)
8	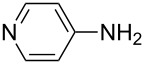 **3h**	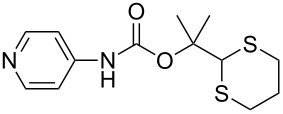 **5h** (52%)	**3h** (48%)
9	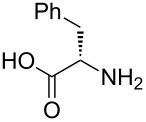 **3i**	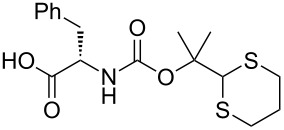 **5i** (80%)	**3i** (41%)

^a^Reaction conditions: For converting **3a**–**d** to **5a**–**d**, **3** (1 equiv), **4** (1 equiv), DIPEA (5 equiv), THF, rt, 8 h. For **3e**–**h** to **5e**–**h**, **3** (1 equiv), **4** (1 equiv), LDA (2 equiv), THF, −78 °C to rt, 8 h. For **3i** to **5i**, **3i** (1 equiv), **4** (1 equiv), DIPEA (5 equiv), DMSO, rt, 8 h. For **5** to **3**, **5** (1 equiv), NaIO_4_ (10 equiv), THF/H_2_O (v/v 1:1), rt, 12 h; then K_2_CO_3_ (10 equiv), MeOH (MeOH/H_2_O for **5i** to **3i**), rt, 1 h. Isolated yields were obtained in all cases except for **3i**, for which the yield was determined with RP-HPLC.

After suitable conditions for protection of amines with dM-Dmoc were identified, we investigated the substrate scope of the reaction. As shown in Table 1, primary aliphatic amines including **3a**–**d** gave good to excellent isolated yields of carbamates **5a**–**d** (Table 1, entries 1–4). Under these conditions, however, secondary aliphatic amines could not react or could react but gave very low yields. We tried a variety of other conditions such as deprotonating the amine followed by reacting with **4** and heating excess amine with **4** without any solvent but failed to identify one that could afford useful yields. We also tried to use the optimized conditions for the protection of aliphatic primary amines to protect arylamines, but found that arylamines were not reactive enough for the reaction. Therefore, for protecting arylamines, we used conditions for the formation of hindered *O*-*tert*-alkyl *N*-arylcarbamates we reported earlier [20]. Treating one equivalent **3e** with two equivalents LDA and one equivalent **4** in THF gave the desired arylamine dM-Dmoc carbamate **5e** in synthetically useful yield (Table 1, entry 5). Three additional arylamines were also tested, which include the two heterocyclic arylamines **3g** and **3h**, all gave synthetically useful yields of the aryl carbamate products **5f**–**h** (Table 1, entries 6–8). Finally, to investigate the suitability of the dM-Dmoc group for protecting amino acids, phenylalanine (**3i**) was selected to react with **4** to give **5i** (Table 1, entry 9). The general conditions for aliphatic amine protection were used, but due to the low solubility of **3i** in THF, DMSO was used as the solvent. Compound **5i** was obtained in 80% isolated yield.

For deprotection of dM-Dmoc protected amines, we used the conditions we developed earlier for the deprotection of Dmoc protected ODNs directly without making additional efforts to evaluate other conditions [18]. These conditions could be superior to reported conditions [13,15,17,21] because they do not require transition metal catalysts or any special devices such as an electrochemical cell. Therefore, the dM-Dmoc carbamates were first oxidized with sodium periodate at room temperature. After removing the excess oxidizing agent and other inorganic salts by filtration, β-elimination to give the amine products was initiated with the weak base potassium carbonate at room temperature. The products were then purified with flash column chromatography. As shown in Table 1, the yields of the deprotection ranged from 48% to 88%. Among them, **3a** and **3d**, which are aliphatic amines, gave better yields (Table 1, entries 1 and 4). Compounds **3b** and **3c** are also aliphatic amines, but their yields were lower. This might be caused by evaporation due to their low boiling points during work-up and purification. The arylamines were obtained in lower yields (Table 1, entries 5–8) compared with the aliphatic ones. Among the four arylamine examples, **5g** and **5h** contained a pyridine ring, which could be sensitive to oxidative conditions. However, it looked like that sodium periodate was benign to pyridine and some other nitrogen containing aromatic heterocycles [18]. The dM-Dmoc protected phenylalanine (**5i**) was deprotected under slightly different conditions (Table 1, entry 9). In the β-elimination step, when methanol was used as the solvent as in the general deprotection procedure, no reaction occurred even after stirring overnight. This might be caused by the more favoured deprotonation of the carboxylic acid group by potassium carbonate, which made the starting material insoluble and prevented deprotonation of H-2 in the oxidized 1,3-dithiane function. The problem was solved by using a solvent mixture of methanol and water. It is important to note that carrying out the deprotection reaction in one pot by performing the oxidation under basic conditions is not feasible. In theory, using the one pot approach, once the sulfides in dM-Dmoc were oxidized, β-elimination would follow to give the desired amine products directly. We tested the idea, and as expected, complex mixtures were formed. Reasons for the observation include oxidation of amine products by sodium periodate and its reduced products. In addition, we also found that oxidation of sulfides by sodium periodate was significantly slower under basic conditions than under neutral and acidic conditions.

**Scheme 2:**
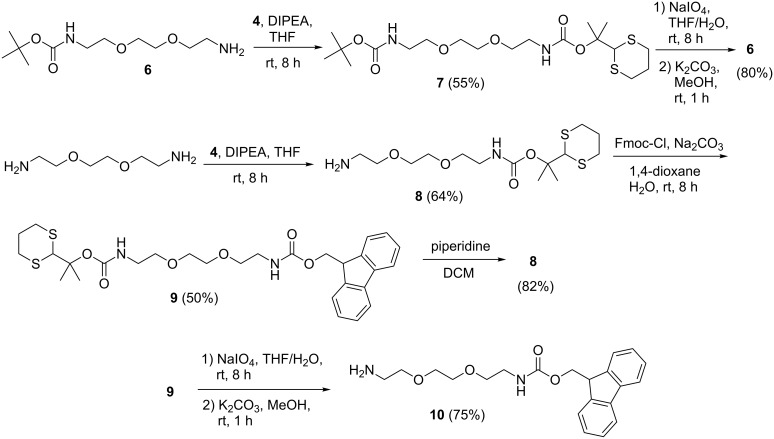
Selective deprotection of dM-Dmoc-, Boc- and Fmoc-protected amines.

To demonstrate the feasibility of selective deprotection of dM-Dmoc protected amines in the presence of Boc protected ones, compound **6** [22] was reacted with **4** under the general aliphatic amine protection conditions to give the Boc and dM-Dmoc protected diamine **7** (Scheme 2). Selective removal of dM-Dmoc was simply achieved under the general deprotection conditions without any fine tuning of conditions. The desired Boc protected **6** was obtained in 80% isolated yield. To demonstrate the orthogonality of dM-Dmoc and Fmoc protections, compound **9** was prepared (Scheme 2). Compound **4** was reacted with 1,2-bis(2-aminoethoxy)ethane to give **8**, which was reacted with Fmoc-Cl to give the dM-Dmoc and Fmoc protected diamine **9**. We found that selective removal of Fmoc from **9** to give **8** could be achieved under typical Fmoc deprotection conditions involving piperidine. Selective removal of dM-Dmoc was also simple; treating **9** under the standard dM-Dmoc deprotection conditions gave the Fmoc protected diamine **10** in 75% isolated yield (Scheme 2). The basic conditions involving potassium carbonate used to induce β-elimination of oxidized dM-Dmoc did not cause any loss of Fmoc protection.

## Conclusion

In summary, we have demonstrated that dM-Dmoc could serve as a new protecting group for aliphatic and arylamines. This group could be removed under nearly neutral oxidative conditions, which are orthogonal to the commonly used conditions for deprotection of protected amines including acid, base, and catalytic hydrogenation. Compared to Dmoc, dM-Dmoc has the advantage of being stable under a wide range of basic and nucleophilic conditions. We expect that the new protecting group will find wide applications in multistep organic synthesis.

## Experimental

**General**: All reactions were performed in oven-dried glassware under an argon atmosphere using standard Schlenk techniques. Reagents and solvents available from commercial sources were used as received unless otherwise noted. THF and CH_2_Cl_2_ were dried using an Innovative Technology Pure-Solv™ system. Acetone, pyridine, and diisopropylamine were distilled over CaH_2_ under nitrogen. Thin-layer chromatography (TLC) was performed using Sigma-Aldrich TLC plates, silica gel 60F-254 over glass support, 0.25 μm thickness. Flash column chromatography was performed using SiliCycle silica gel, particle size 40–63 μm. ^1^H and ^13^C spectra were measured on Varian UNITY INOVA spectrometer at 400 and 100 MHz, respectively; chemical shifts (δ) were reported in reference to solvent peaks (residue CHCl_3_ at δ 7.24 ppm for ^1^H and CDCl_3_ at δ 77.00 ppm for ^13^C).

**2-(1,3-Dithian-2-yl)propan-2-ol:** To a solution of 1,3-dithiane (5.0 g, 41.6 mmol) in dry THF (100 mL) was slowly added *n-*BuLi (2.5 M in pentane, 15.7 mL, 41.6 mmol) at −78 °C under argon. After stirring for 30 min, freshly distilled acetone (3.0 mL, 41.6 mmol) was added dropwise, and stirring was continued for 1 h. The reaction was quenched with sat. NH_4_Cl (75 mL) and extracted with EtOAc (50 mL × 2). The extracts were dried over anhydrous Na_2_SO_4_, filtered, and concentrated. The residue was purified by flash column chromatography (SiO_2_, 4:1 hexanes/EtOAc) to afford the title compound as a white amorphous solid (6.24 g, 84%): TLC *R*_f_ = 0.3 (4:1 hexanes/EtOAc); IR (thin film) *ν* 3339, 2930, 1420 cm^−1^; ^1^H NMR (400 MHz, CDCl_3_) δ 1.32 (s, 6H), 1.73–1.85 (m, 1H), 2.00–2.07 (m, 1H), 2.41 (s, 1H), 2.78–2.90 (m, 4H), 4.10 (s, 1H) ppm; ^13^C NMR (100 MHz, CDCl_3_) δ 25.9, 27.4, 30.9, 61.0, 73.4 ppm; HRMS (ESI) *m*/*z*: [M + K]^+^ calcd for C_7_H_14_OS_2_K, 217.0123; found, 217.0121.

**2-(1,3-Dithian-2-yl)propan-2-yl (4-nitrophenyl) carbonate (4):** To a solution of 2-(1,3-dithian-2-yl)propan-2-ol (6.4 g, 36 mmol, 1 equiv) and pyridine (2.9 mL, 54 mmol, 1.5 equiv) in DCM (100 mL) was added *p*-nitrophenylchloroformate (7.2 g, 36 mmol, 1 equiv) at rt under argon. After stirring for 8 h, the contents were poured into a separatory funnel and partitioned between EtOAc (40 mL) and H_2_O (80 mL). The aqueous layer was extracted with DCM (50 mL × 2). The combined organic layer was dried over anhydrous Na_2_SO_4_, filtered and concentrated. Flash column chromatography (SiO_2_, 9:1 hexanes/EtOAc) gave **4** as a white amorphous solid (10.0 g, 81%): TLC *R*_f_ = 0.4 (5:1 hexanes/EtOAc); IR (thin film) *ν* 3083, 2981, 1713, 1592, 1522 cm^−1^; ^1^H NMR (400 MHz, CDCl_3_) δ 1.70 (s, 6H), 1.81–1.91 (m, 1H), 2.11–2.18 (m, 1H), 2.92–2.95 (m, 4H), 4.98 (s, 1H), 7.38 (d, *J* = 9.2 Hz, 2H), 8.26 (d, *J* = 6.9 Hz, 2H) ppm; ^13^C NMR (100 MHz, CDCl_3_) δ 24.1, 25.7, 30.8, 56.2, 86.9, 121.9, 125.1, 145.2, 150.0, 155.5 ppm; HRMS (ESI) *m*/*z*: [M + K]^+^ calcd for C_14_H_18_O_2_S_2_K, 321.0385; found, 321.0404.

**General procedure for dM-Dmoc protection of aliphatic amines – synthesis of carbamates 5a**–**d:** To a solution of an amine (0.292 mmol, 1 equiv) and DIPEA (0.255 mL, 1.46 mmol, 5 equiv) in dry THF (10 mL) was added **4** (0.100 g, 0.292 mmol, 1 equiv) at rt under argon. After stirring for 8 h, the reaction was quenched with sat. NH_4_Cl (15 mL). Organics were extracted with EtOAc (10 mL × 2). The extracts were dried over anhydrous Na_2_SO_4_, filtered, and concentrated. The carbamates **5a** (column eluted with 3:1 hexanes/EtOAc; TLC *R*_f_ = 0.5, developed with 1:1 hexanes/EtOAc), **5b** (6:1 hexanes/EtOAc; *R*_f_ = 0.5, 3:1 hexanes/EtOAc), **5c** (6:1 hexanes/EtOAc; *R*_f_ = 0.5, 3:1 hexanes/EtOAc), and **5d** (9:1 hexanes/EtOAc; *R*_f_ = 0.5, 6:1 hexanes/EtOAc) were purified with flash column chromatography (SiO_2_).

**2-(1,3-Dithian-2-yl)propan-2-yl benzylcarbamate (5a):** Colorless oil (0.084 g, 92%); IR (thin film) *ν* 3343, 3020, 2937, 1697, 1506, 1452 cm^−1^; ^1^H NMR (400 MHz, CDCl_3_) δ 1.58 (s, 6H), 1.75–1.84 (m, 1H), 2.04–2.09 (m, 1H), 2.81–2.92 (m, 4H), 4.30 (d, *J* = 5.88 Hz, 2H), 5.00 (brs, 1H), 5.09 (s, 1H), 7.23–7.31 (m, 5H) ppm; ^13^C NMR (100 MHz, CDCl_3_) δ 25.1, 26.2, 31.2, 44.8, 57.6, 82.4, 127.5, 127.6, 128.7, 138.8, 155.3 ppm; HRMS (ESI) *m*/*z*: [M + Na]^+^ calcd for C_15_H_21_NO_2_S_2_Na, 334.0911; found, 334.0905.

**2-(1,3-Dithian-2-yl)propan-2-yl butylcarbamate (5b):** Pale yellow oil (0.072 g, 89%); IR (thin film) *ν* 3359, 2930, 1710, 1516 cm^−1^; ^1^H NMR (400 MHz, CDCl_3_) δ 0.87 (t, *J* = 7.24 Hz, 3H), 1.27–1.36 (m, 2H), 1.43–1.45 (m, 2H), 1.54 (s, 6H), 1.75–1.82 (m, 1H), 2.02–2.08 (m, 1H), 2.79–2.92 (m, 4H), 3.06–3.11 (m, 2H), 4.62 (brs, 1H), 5.04 (s, 1H) ppm; ^13^C NMR (100 MHz, CDCl_3_) δ 13.9, 20.0, 25.1, 26.2, 31.2, 32.1, 40.6, 57.7, 81.9, 155.3 ppm; HRMS (ESI) *m*/*z*: [M + Na]^+^ calcd for C_12_H_23_NO_2_S_2_Na, 300.1068; found, 300.1056.

**2-(1,3-Dithian-2-yl)propan-2-yl allylcarbamate (5c):** Orange solid (0.055 g, 72%). mp 67–68 °C; IR (thin film) *ν* 3343, 3080, 2940, 1707, 1516 cm^−1^; ^1^H NMR (400 MHz, CDCl_3_) δ 1.54 (s, 6H), 1.70–1.81 (m, 1H), 1.99–2.08 (m, 1H), 2.79–2.91 (m, 4H), 3.70–3.72 (m, 2H), 4.76 (brs, 1H), 5.05–5.08 (m, 2H), 5.17 (d, *J* = 17.3 Hz, 1H), 5.74–5.84 (m, 1H) ppm; ^13^C NMR (100 MHz, CDCl_3_) δ 25.1, 26.2, 31.2, 43.2, 57.5, 82.2, 116.0, 134.7, 155.1 ppm; HRMS (ESI) *m*/*z*: [M + K]^+^ calcd for C_11_H_19_NO_2_S_2_K, 300.0494; found, 300.0496.

**2-(1,3-Dithian-2-yl)propan-2-yl (2-((*tert*-butyldiphenylsilyl)oxy)ethyl)carbamate (5d):** Colorless oil (0.142 g, 97%); IR (thin film) *ν* 3369, 3076, 2930, 1713, 1510, 1452 cm^−1^; ^1^H NMR (400 MHz, CDCl_3_) δ 1.04 (s, 9H), 1.57 (s, 6H), 1.74–1.83 (m, 1H), 2.04–2.11 (m, 1H), 2.82–2.92 (m, 4H), 3.27–3.31 (m, 2H), 3.69–3.71 (m, 2H), 4.96 (brs, 1H), 5.07 (s, 1H), 7.34–7.41 (m, 6H), 7.62–7.64 (m, 4H) ppm; ^13^C NMR (100 MHz, CDCl_3_) δ 19.4, 25.1, 26.2, 27.0, 31.1, 43.1, 57.6, 63.2, 82.2, 127.7, 129.9, 133.5, 135.7, 155.3 ppm; HRMS (ESI) *m*/*z*: [M + Na]^+^ calcd for C_26_H_37_NO_3_S_2_SiNa, 526.1882; found, 526.1875.

**General procedure for dM-Dmoc protection of arylamines – synthesis of carbamates 5e**–**h:** To a solution of diisopropylamine (0.076 mL, 0.541 mmol, 2.1 equiv) in THF (10 mL) at −78 °C under argon was added *n-*BuLi (2.5 M in pentane, 0.206 mL, 0.514 mmol, 2 equiv). The mixture was stirred for 15 min. To the freshly prepared LDA solution was added an amine (0.257 mmol, 1 equiv) in THF (50 mL) at −78 °C. After stirring for 45 min, solid **4** (0.088 g, 0.257 mmol, 1 equiv) was added to the amide solution at −78 °C under positive argon pressure. The mixture was stirred for 8 h while warming to rt gradually. The reaction was quenched with sat. NH_4_Cl (15 mL) and extracted with EtOAc (10 mL × 2). The extracts were dried over anhydrous Na_2_SO_4_, filtered, and concentrated. The carbamates **5e** (column eluted with 9:1 hexanes/EtOAc; TLC *R*_f_ = 0.2, developed with 5:1 hexanes/EtOAc), **5f** (6:1 hexanes/EtOAc; *R*_f_ = 0.4, 3:1 hexanes/EtOAc), **5g** (5:1 hexanes/EtOAc; *R*_f_ = 0.5, 2:1 hexanes/EtOAc), and **5h** (98:2 EtOAc/MeOH; *R*_f_ = 0.5, 9:1 EtOAc/MeOH) were purified with flash column chromatography (SiO_2_).

**2-(1,3-Dithian-2-yl)propan-2-yl phenylcarbamate (5e):** Brown oil (0.035 g, 46%); IR (thin film) *ν* 3359, 3002, 2927, 1780, 1592, 1487 cm^−1^; ^1^H NMR (400 MHz, CDCl_3_) δ 1.43 (s, 6H), 1.68–1.77 (m, 1H), 2.02–2.08 (m, 1H), 2.80–2.91 (m, 4H), 5.09 (s, 1H), 7.21–7.33 (m, 4H) ppm; ^13^C NMR (100 MHz, CDCl_3_) δ 24.6, 26.1, 31.1, 56.3, 85.3, 127.8, 128.2, 128.6, 138.7, 151.0 ppm; HRMS (ESI) *m*/*z*: [M + K]^+^ calcd for C_14_H_19_NO_2_S_2_K, 336.0754; found, 336.0760.

**2-(1,3-Dithian-2-yl)propan-2-yl (4-nitrophenyl)carbamate (5f):** Brown oil (37 mg, 42%); IR (thin film) *ν* 3305, 3080, 2971, 1732, 1620, 1595 cm^−1^; ^1^H NMR (400 MHz, CDCl_3_) δ 1.65 (s, 6H), 1.78–1.85 (m, 1H), 2.08–2.12 (m, 1H), 2.85–2.92 (m, 4H), 5.02 (s, 1H), 6.95 (brs, 1H), 7.50 (d, *J* = 9.2 Hz, 2H), 8.17 (d, *J* = 9.2 Hz, 2H) ppm; ^13^C NMR (100 MHz, CDCl_3_) δ 25.1, 26.2, 31.3, 57.4, 84.6, 117.8, 125.3, 143.0, 144.1, 151.2 ppm; HRMS (ESI) *m*/*z*: [M + Na]^+^ calcd for C_14_H_18_N_2_O_4_S_2_Na, 365.0605; found, 365.0610.

**2-(1,3-Dithian-2-yl)propan-2-yl (pyridin-2-yl)carbamate (5g):** Pale brown oil (0.043 g, 57%); IR (thin film) *ν* 3184, 2930, 1719, 1640, 1583 cm^−1^; ^1^H NMR (400 MHz, CDCl_3_) δ 1.63 (s, 6H), 1.75–1.82 (m, 1H), 2.02–2.08 (m, 1H), 2.80–2.88 (m, 4H), 5.14 (s, 1H), 6.90–6.92 (m, 1H), 7.61–7.65 (m, 1H), 7.93 (d, *J* = 8.4 Hz, 1H), 8.33–8.35 (m, 1H), 9.70 (brs, 1H) ppm; ^13^C NMR (100 MHz, CDCl_3_) δ 25.3, 26.2, 31.3, 57.2, 83.5, 112.8, 118.4, 138.4, 147.8, 152.3, 152.5 ppm; HRMS (ESI) *m*/*z*: [M + Na]^+^ calcd for C_13_H_18_N_2_O_2_S_2_Na, 321.0707; found, 321.0701.

**2-(1,3-Dithian-2-yl)propan-2-yl (pyridin-4-yl)carbamate (5h):** Orange oil (0.040 g, 52%); IR (thin film) *ν* 3170, 2950, 1719, 1636, 1580 cm^−1^; ^1^H NMR (400 MHz, CDCl_3_) δ 1.63 (s, 6H), 1.76–1.84 (m, 1H), 2.06–2.12 (m, 1H), 2.84–2.91 (m, 4H), 5.01 (s, 1H), 7.39 (d, *J* = 6.5 Hz, 2H), 7.55 (brs, 1H), 8.39 (d, *J* = 6.2 Hz, 2H) ppm; ^13^C NMR (100 MHz, CDCl_3_) δ 24.9, 26.1, 31.3, 57.3, 84.5, 112.9, 146.8, 149.2, 151.3 ppm; HRMS (ESI) *m*/*z*: [M + H]^+^ calcd for C_13_H_18_N_2_O_2_S_2_H, 299.0887; found, 299.0879.

***N*-(((2-(1,3-Dithian-2-yl)propan-2-yl)oxy)carbonyl)-L-phenylalanine (5i)**: This compound was prepared following the general procedure for dM-Dmoc protection of aliphatic amines with slight modifications: DMSO instead of THF was used as the solvent. During work-up, 5% citric acid (instead of sat. NH_4_Cl) and EtOAc were used for partition. Flash column chromatography (SiO_2_, 3:1:0.04 hexanes/EtOAc/AcOH) gave **5i** as a white foam after co-evaporation with toluene (80%): TLC *R*_f_ = 0.2 (1:1:0.02 hexanes/EtOAc/AcOH); IR (thin film) *ν* 3331, 3035, 2933, 1715, 1497 cm^−1^; ^1^H NMR (400 MHz, CDCl_3_) two rotamers, δ 1.43 (s, 1.2H), 1.45 (s, 1.2H), 1.51 (s, 1.8H), 1.55 (s, 1.8H), 1.74–1.79 (m, 1H), 2.03–2.07 (m, 1H), 2.84–2.87 (m, 4H), 2.98–3.08 (m, 1H), 3.15–3.20 (m, 1H), 4.44–4.50 (m, 0.4H), 4.57–4.62 (m, 0.6H), 4.84 (s, 0.4H), 5.02 (s, 0.6H), 5.17–5.18 (m, 1H), 6.47 (bs, 1H), 7.18–7.28 (m, 5H) ppm; ^13^C NMR (100 MHz, CDCl_3_) two rotamers, δ 24.8, 25.1, 26.2, 31.2, 38.1, 39.3, 54.6, 57.5, 83.2, 84.3, 127.2, 128.7, 129.7, 135.8, 154.5, 155.6, 175.9, 176.3 ppm; HRMS (ESI) *m*/*z*: [M − H]^−^ calcd for C_17_H_22_NO_4_S_2_, 368.0996; found, 368.0987.

**General procedure for deprotection of dM-Dmoc protected amines:** To a suspension of **5** (1 equiv) in THF/H_2_O (v/v 1:1) was added NaIO_4_ (10 equiv) at rt. After stirring overnight, the mixture was concentrated on a rotary evaporator. The residue was dissolved in acetone (5% AcOH in acetone for **5i**), and the insoluble inorganic salts were removed by filtration. The filtrate was concentrated on a rotary evaporator. The residue (after co-evaporation with toluene for **5i**) was suspended in methanol (dissolved in 1:1 methanol/H_2_O in the case of **5i**). Finely ground K_2_CO_3_ (10 equiv) was added, and the mixture was stirred at rt for 1 h. Insoluble salts were removed by filtration. The filtrate was concentrated on a rotary evaporator and purified via flash column chromatography (SiO_2_). All amine products **3a**–**i** were known and were indentified with TLC and NMR. Chromatography and TLC information: **3a** (column eluted with 8.5:1:0.5 EtOAc/MeOH/Et_3_N; TLC *R*_f_ = 0.4, 9:1 EtOAc/MeOH), **3b** (8.5:1:0.5 EtOAc/MeOH/Et_3_N; *R*_f_ = 0.2, 9:1 EtOAc/MeOH), **3c** (8.5:1:0.5 EtOAc/MeOH/Et_3_N; *R*_f_ = 0.2, 9:1 EtOAc/MeOH), **3d** (9:0.5:0.5 EtOAc/MeOH/Et_3_N; *R*_f_ = 0.5, 9:1 EtOAc/MeOH), **3e** (9:1 hexanes/EtOAc; *R*_f_ = 0.2, 5:1 hexanes/EtOAc), **3f** (5:1 hexanes/EtOAc; *R*_f_ = 0.3, 3:1 hexanes/EtOAc), **3g** (5:1 hexanes/EtOAc; *R*_f_ = 0.4, 2:1 hexanes/EtOAc), **3h** (9.5:0.5 EtOAc/MeOH; *R*_f_ = 0.5, 9:1 EtOAc/MeOH), **3i** (*R*_f_ = 0.5, 3:1:1 *n-*BuOH/AcOH/H_2_O). Isolated yields were obtained for **3a**–**h** (see Table 1). Yield of **3i** was determined to be 41% using RP-HPLC (column: C-18, 5 μm, 100 Å, 250 × 3.20 mm; solvent A: 0.1 M triethylammonium acetate, 5% acetonitrile; solvent B: 90% acetonitrile; gradient: time, 0–60 min, solvent B, 0–45%; flow rate: 1 mL/min; detection: UV 257 nm) by comparing with authentic sample.

**Selective deprotection of dM-Dmoc protected amine in the presence of a Boc protected amine:** Compound **6** was prepared following a reported procedure [22]. The general procedure for dM-Dmoc protection of aliphatic amines (i.e., the procedure for the synthesis of **5a**–**d**) was used to covert **6** to **7**. Compound **7** was purified with flash column chromatography (SiO_2_, 1:1 hexanes/EtOAc): Colorless oil (55%); TLC *R*_f_ = 0.2 (1:1 hexanes/EtOAc); IR (thin film) *ν* 3356, 2933, 1707, 1690, 1510 cm^−1^; ^1^H NMR (400 MHz, CDCl_3_) δ 1.41 (s, 9H), 1.54 (s, 6H), 1.74–1.81 (m, 1H), 2.03–2.09 (m, 1H), 2.83–2.88 (m, 4H), 3.28–3.32 (m, 4H), 3.50–3.53 (m, 4H), 3.57 (s, 4H), 5.04 (s, 1H) ppm; ^13^C NMR (100 MHz, CDCl_3_) δ 25.1, 26.2, 28.6, 31.1, 40.7, 57.6, 70.4, 79.5, 82.2, 155.4, 156.2 ppm; HRMS (ESI) *m*/*z*: [M + Na]^+^ calcd for C_19_H_36_N_2_O_6_S_2_Na, 475.1912; found, 475.1898. Selective removal of dM-Dmoc in the presence of Boc in **7** to give **6** was achieved following the general procedure for deprotection of dM-Dmoc protected amines. The product was purified by flash column chromatography (SiO_2_, 9.5:0.5 DCM/MeOH; TLC *R*_f_ = 0.4, 9:1 DCM/MeOH). Isolated yield of 80% was obtained.

**Selective deprotection of dM-Dmoc and Fmoc protected amine:** The dM-Dmoc and Fmoc protected diamine **9** was prepared from 1,2-bis(2-aminothoxy)ethane. Compound **8** was synthesized using the general procedure for dM-Dmoc protection of aliphatic amines and purified with flash column chromatography (SiO_2_, 9:0.5:0.5 DCM/MeOH/Et_3_N): Light yellow oil (64%); TLC *R*_f_ = 0.3 (9:1 DCM/MeOH); IR (thin film) *ν* 3359, 2937, 1713, 1519 cm^−1^; ^1^H NMR (400 MHz, CDCl_3_) δ 1.51 (s, 6H), 1.69–1.76 (m, 1H), 2.00–2.06 (m, 1H), 2.80–2.89 (m, 4H), 3.05 (bs, 2H), 3.25 (bs, 2H), 3.50 (t, J = 4.7 Hz, 4H), 3.56 (s, 4H) ppm, 5.00 (s, 1H), 5.44 (bs, 1H); ^13^C NMR (100 MHz, CDCl_3_) δ 25.2, 26.3, 31.2, 40.7, 41.5, 57.7, 70.2, 70.3, 70.4, 82.1, 155.3 ppm; HRMS (ESI) *m*/*z*: [M + H]^+^ calcd for C_14_H_28_N_2_O_4_S_2_H, 353.1569; found, 353.1576. For the synthesis of **9**, compound **8** (0.225 g, 0.640 mmol, 1 equiv) in 1,4-dioxane (5 mL) and 15% Na_2_CO_3_ (5 mL) was reacted with Fmoc-Cl (0.402 g, 0.292 mmol, 1 equiv) at rt under argon. After 8 h, the reaction mixture was diluted with EtOAc (50 mL), and the organic and aqueous phases were separated. The former was washed with water (25 mL), 5% citric acid (25 mL) and brine (25 mL × 2), dried over anhydrous Na_2_SO_4_, filtered, and concentrated to dryness. The residue was purified with flash column chromatography (SiO_2_, 1:1 hexanes/EtOAc): Light yellow oil (0.185 g, 50%); TLC *R*_f_ = 0.2 (1:1 hexanes/EtOAc); IR (thin film) *ν* 3337, 3070, 2943, 1713, 1449 cm^−1^; ^1^H NMR (400 MHz, CDCl_3_) δ 1.45 (s, 6H), 1.69–1.79 (m, 1H), 2.00–2.04 (m, 1H), 2.77–2.85 (m, 1H), 3.26–3.29 (m, 2H), 3.36–3.37 (m, 2H), 3.49–3.53 (m, 4H), 3.56 (s, 4H), 4.17–4.20 (m, 1H), 4.37–4.38 (m, 2H), 5.02 (m, 1H), 5.16 (bs, 1H), 5.36 (bs, 1H), 7.27 (t, J = 7.4 Hz, 2H), 7.35 (t, J = 7.4 Hz, 2H), 7.56 (d, J = 6.7 Hz, 2H), 7.72 (d, J = 7.4 Hz, 2H) ppm; ^13^C NMR (100 MHz, CDCl_3_) δ 25.2, 26.3, 31.2, 40.7, 41.2, 47.5, 57.7, 68.8, 70.3, 70.5, 82.2, 120.1, 125.2, 127.1, 127.8, 141.4, 144.1, 155.3, 156.6 ppm; HRMS (ESI) *m*/*z*: [M + H]^+^ calcd for C_29_H_38_N_2_O_6_S_2_H, 575.2250; found, 575.2262. For selective removal of the Fmoc group of **9** to give **8**, **9** (90 mg, 0.157 mmol, 1 equiv) in dry DCM (10 mL) was reacted with piperidine (2 mL) at rt under argon for 2 h. The reaction mixture was concentrated to dryness under reduced pressure. The residue was purified with flash column chromatography (SiO_2_, 9:0.5:0.5 DCM/MeOH/Et_3_N; TLC *R*_f_ = 0.3, 9:1 DCM/MeOH) to give **8** as a light yellow oil (45 mg, 82%). For selective removal of the dM-Dmoc group of **9** to give **10** [23], the general procedure for deprotection of dM-Dmoc protected amines was used. The product was purified with flash column chromatography (SiO_2,_ 9:0.5:0.5 DCM/MeOH/Et_3_N, TLC *R*_f_ = 0.5, 8:1:1 DCM/MeOH/Et_3_N). An isolated yield of 75% was obtained.

## Supporting Information

File 1 Images of ^1^H and ^13^C NMR spectra of new compounds including **5a**–**i** and **7**–**9**.
