# Supplementary material for: An amine protecting group deprotectable under nearly neutral oxidative conditions
Source: Beilstein J Org Chem. 2018 Jul 13;14:1750–7. doi: 10.3762/bjoc.14.149 (PMC6071699; doi:10.3762/bjoc.14.149)
Supplement: File 1 — Images of 1H and 13C NMR spectra of new compounds including 5a–i and 7–9. [file Beilstein_J_Org_Chem-14-1750-s001.pdf]

Supporting Information

for

An amine protecting group deprotectable under nearly neutral oxidative conditions

Shahien Shahsavari[1], Chase McNamara[1], Mark Sylvester[1], Emily Bromley[1], Savannah Joslin[1], Bao-Yuan Lu[2] and Shiyue Fang*[1]

Address: [1]Department of Chemistry, Michigan Technological University, 1400 Townsend Drive, Houghton, MI 49931, USA and [2]Nalco Champion, an Ecolab Company, 11177 S. Stadium Drive, Sugar Land, TX 77478, USA

Email: Shiyue Fang - shifang@mtu.edu

*Corresponding author

Images of ^{1}H and ^{13}C NMR spectra of new compounds including 5a–i and 7–9

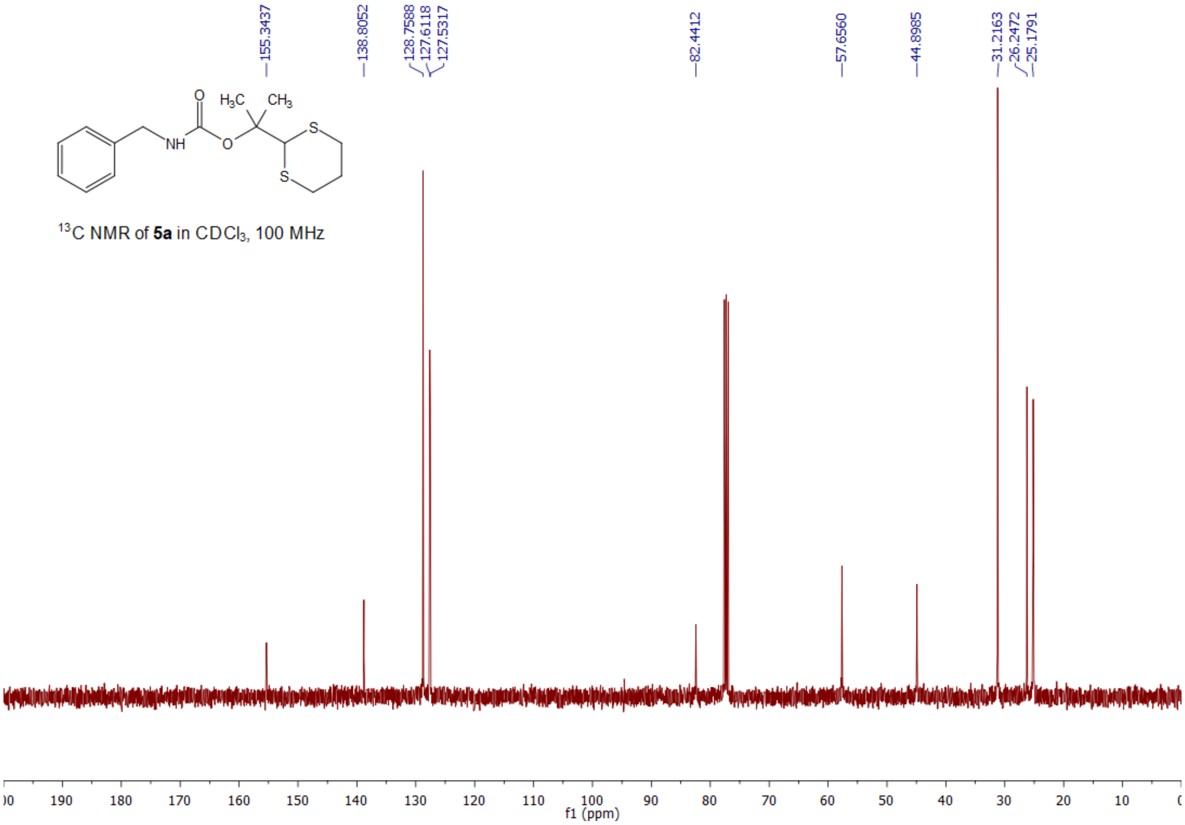

^{13}C NMR of **5a** in $CDCl_3$, 100 MHz

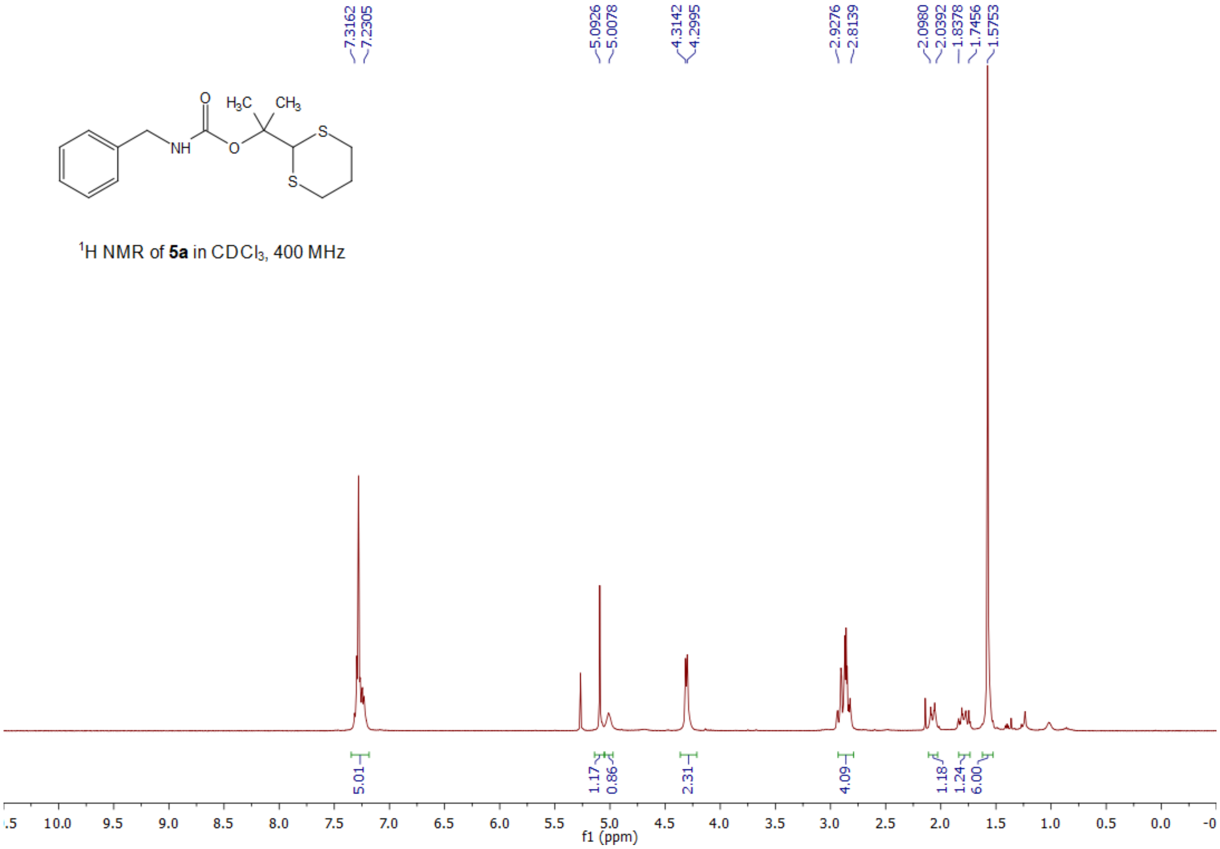

^{1}H NMR of **5a** in $CDCl_3$, 400 MHz

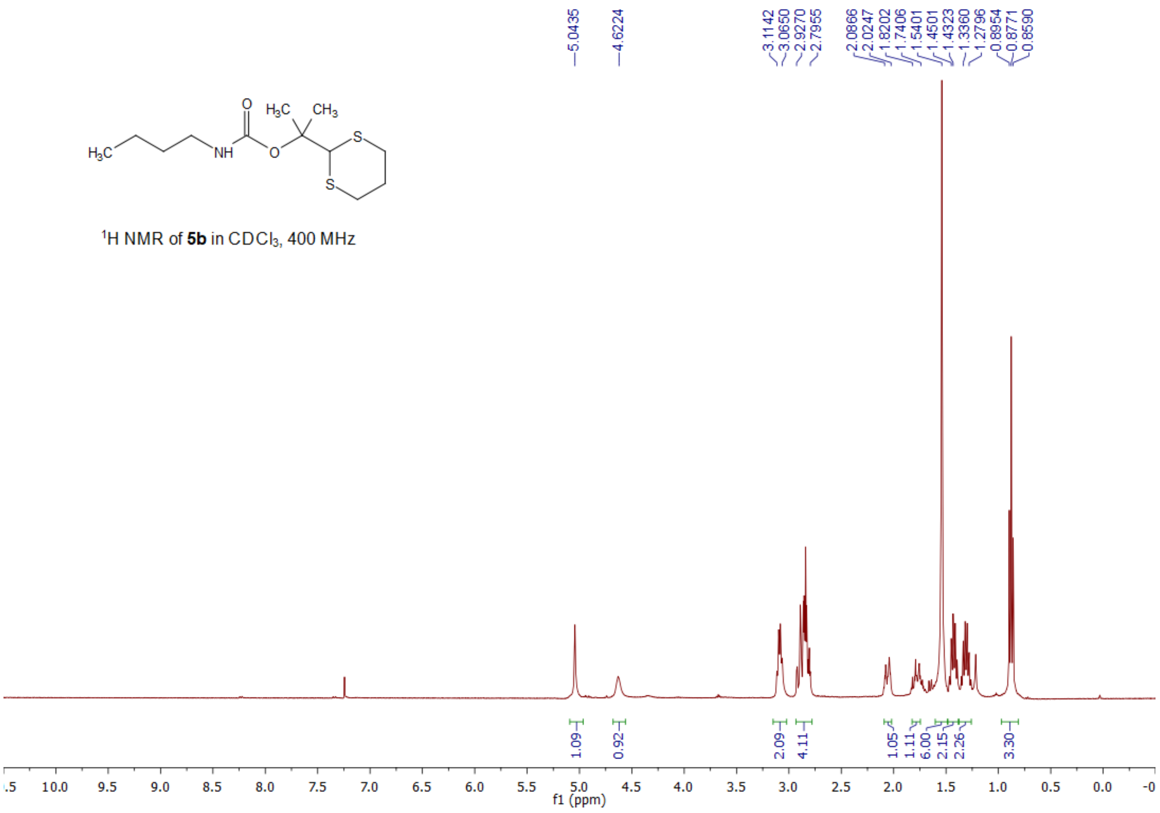
5.0435
4.6224
3.1142
3.0650
2.9270
2.7955
2.0866
2.0247
1.8202
1.7406
1.5401
1.4501
1.4323
1.3360
1.2796
0.8954
0.8771
0.8590
O
H3C
CH3
H3C
NH
O
S
S
1H NMR of 5b in CDCl3, 400 MHz
1.09
0.92
2.09
4.11
1.05
1.11
6.00
2.15
2.26
3.30
f1 (ppm)

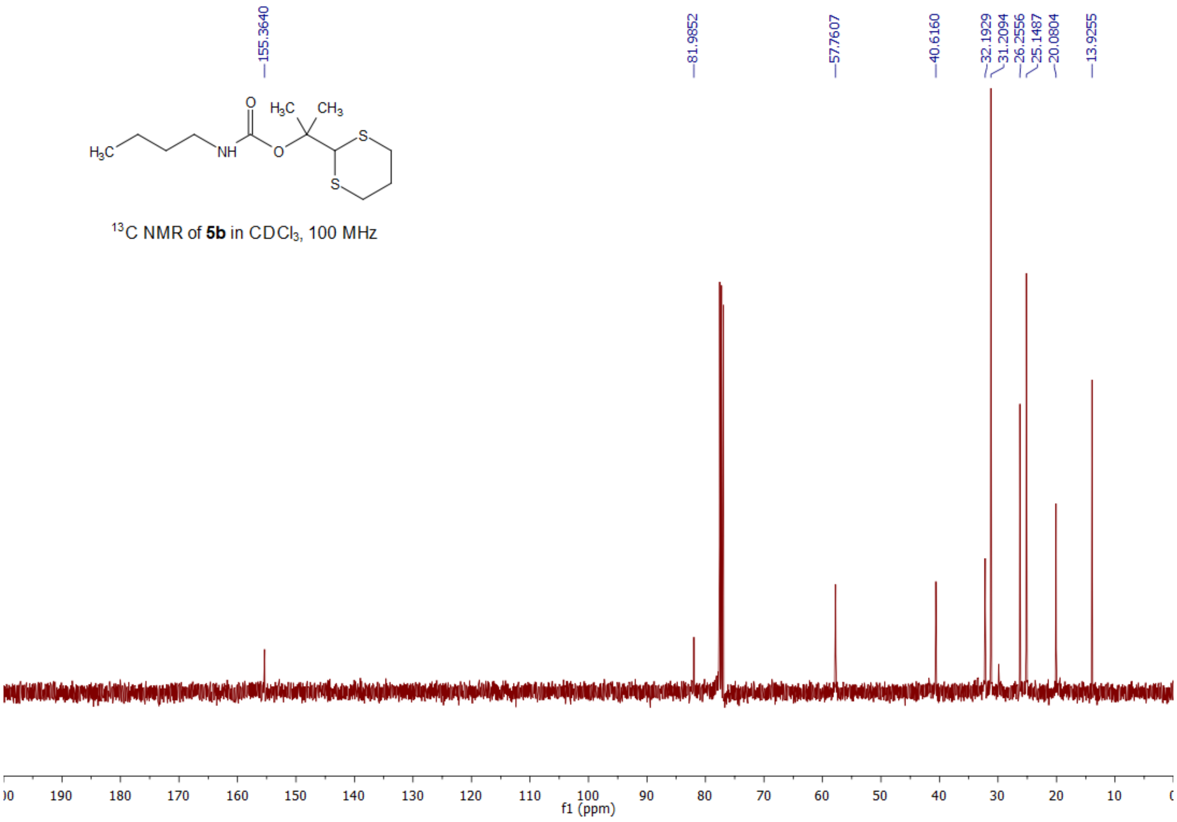
155.3640
81.9852
57.7607
40.6160
32.1929
31.2094
26.2556
25.1487
20.0804
13.9255
O
H3C
CH3
H3C
NH
O
S
S
13C NMR of 5b in CDCl3, 100 MHz
f1 (ppm)

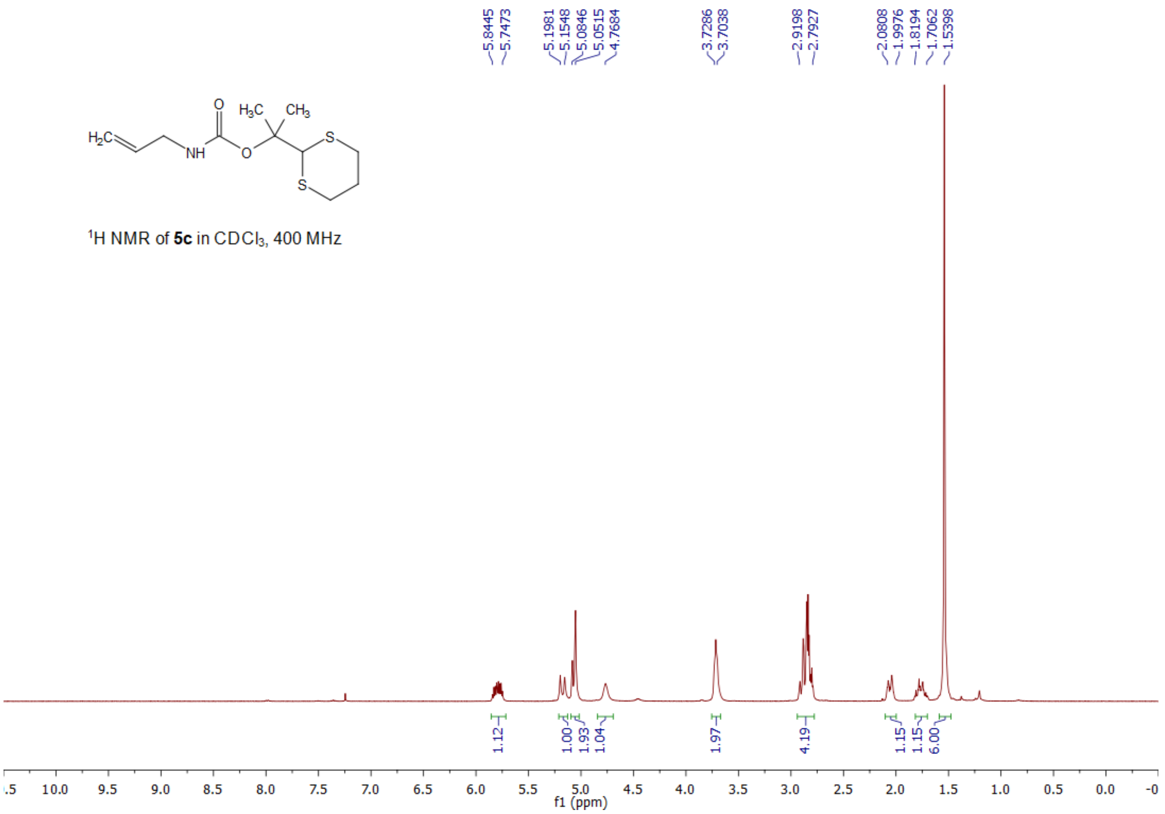

^{1}H NMR of **5c** in $CDCl_3$, 400 MHz

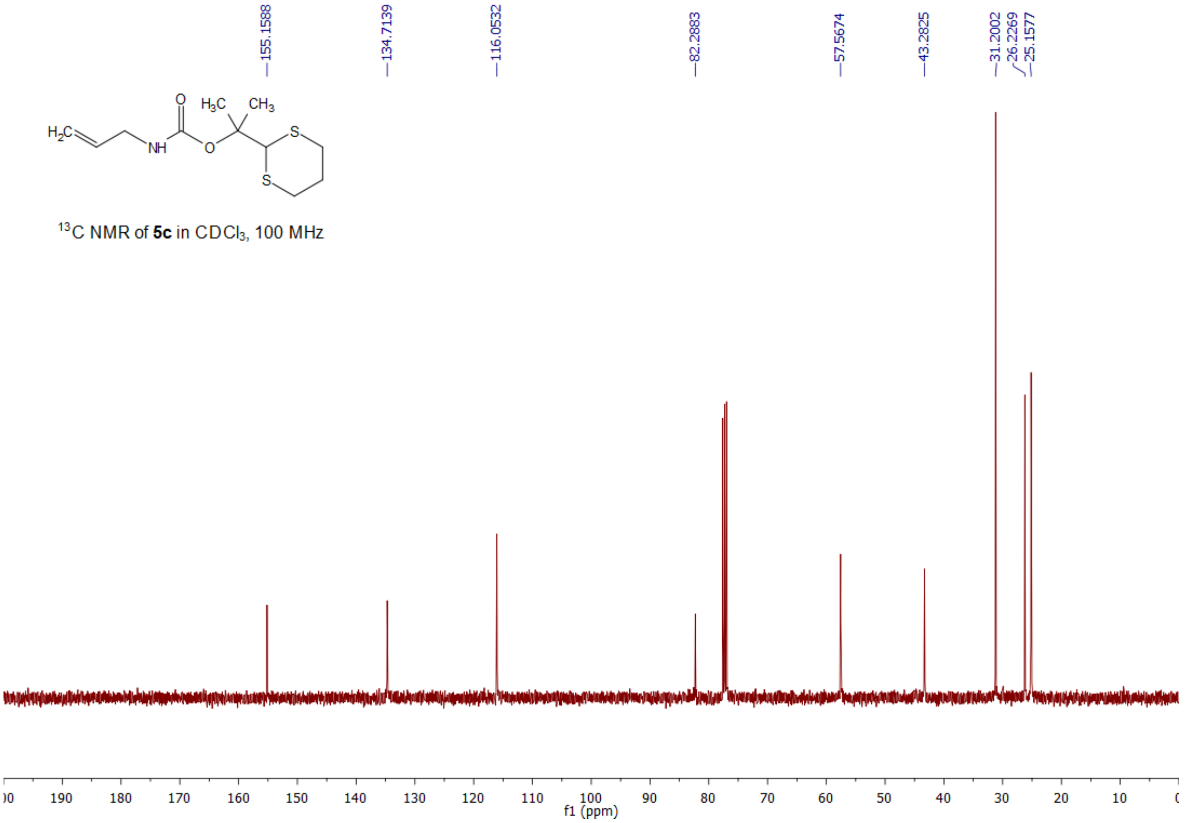

^{13}C NMR of **5c** in $CDCl_3$, 100 MHz

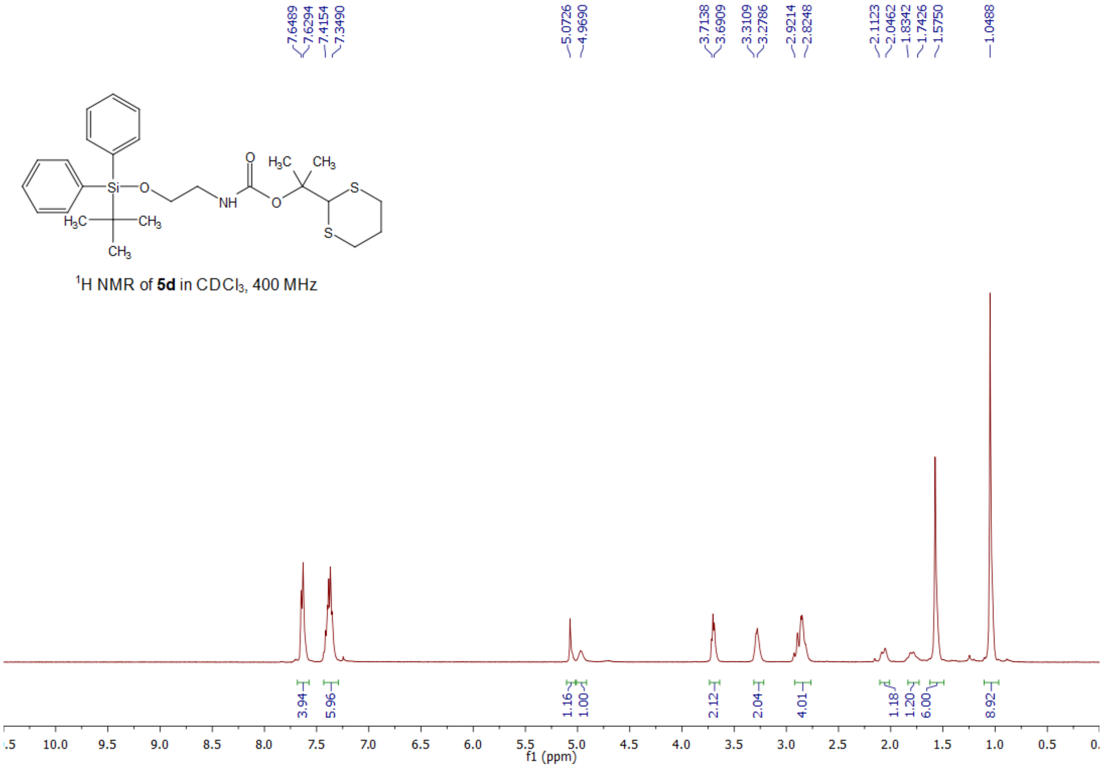

^{1}H NMR of **5d** in $CDCl_3$, 400 MHz

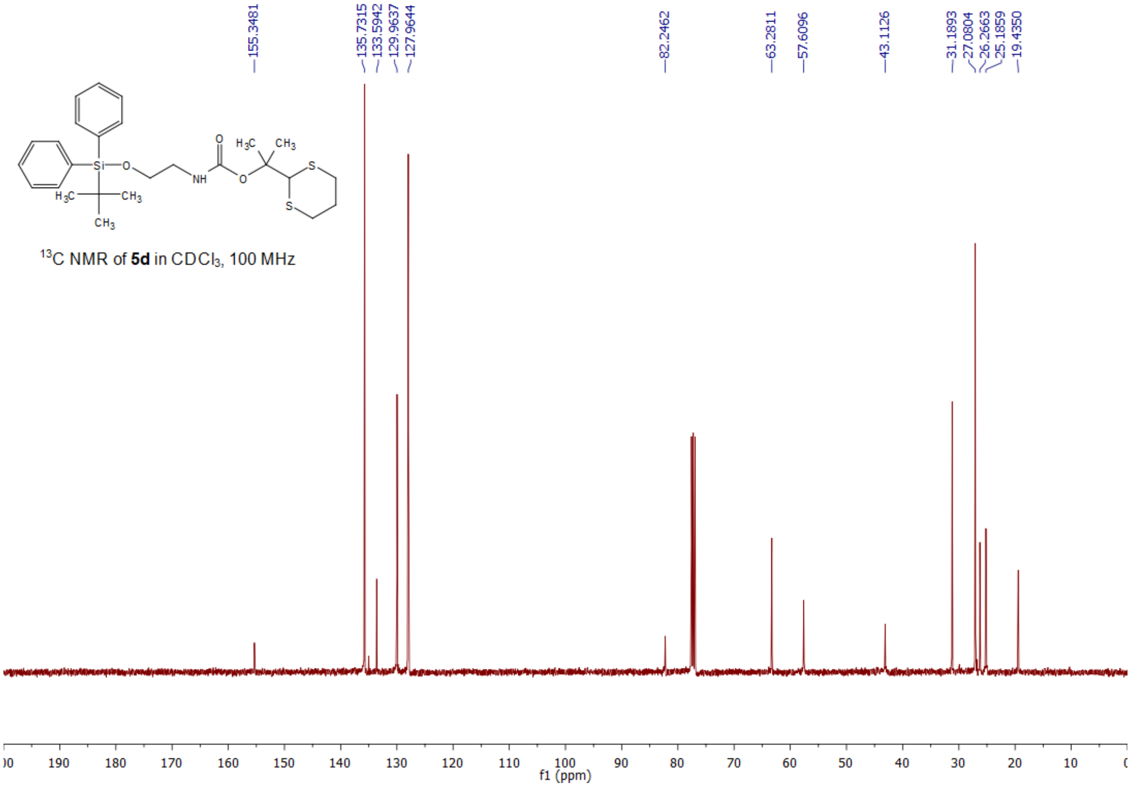

^{13}C NMR of **5d** in $CDCl_3$, 100 MHz

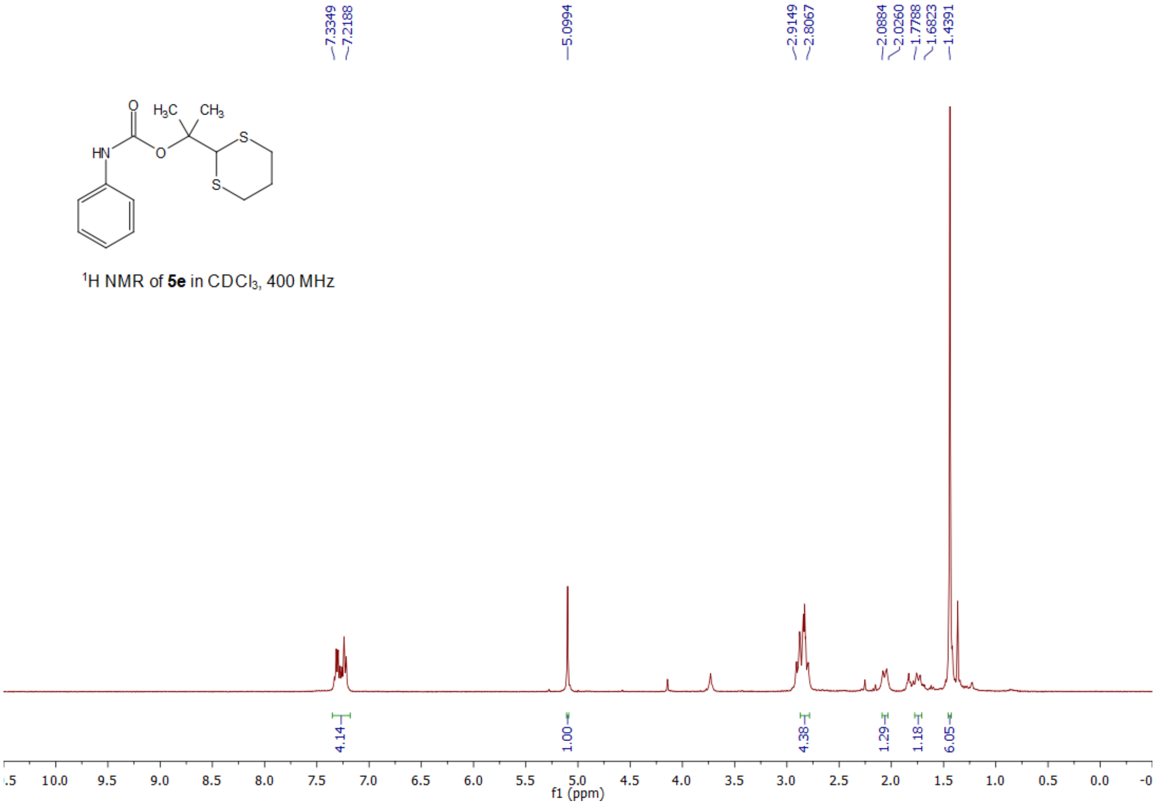

^{1}H NMR of **5e** in $CDCl_3$, 400 MHz

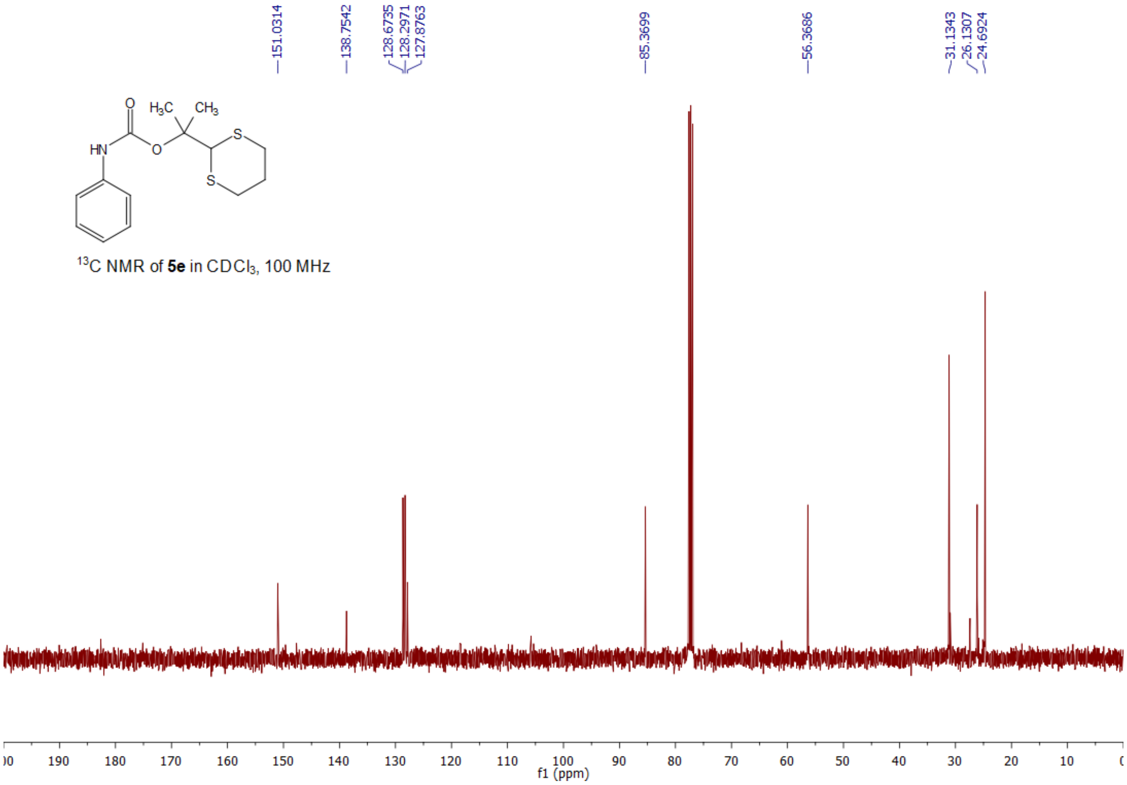

^{13}C NMR of **5e** in $CDCl_3$, 100 MHz

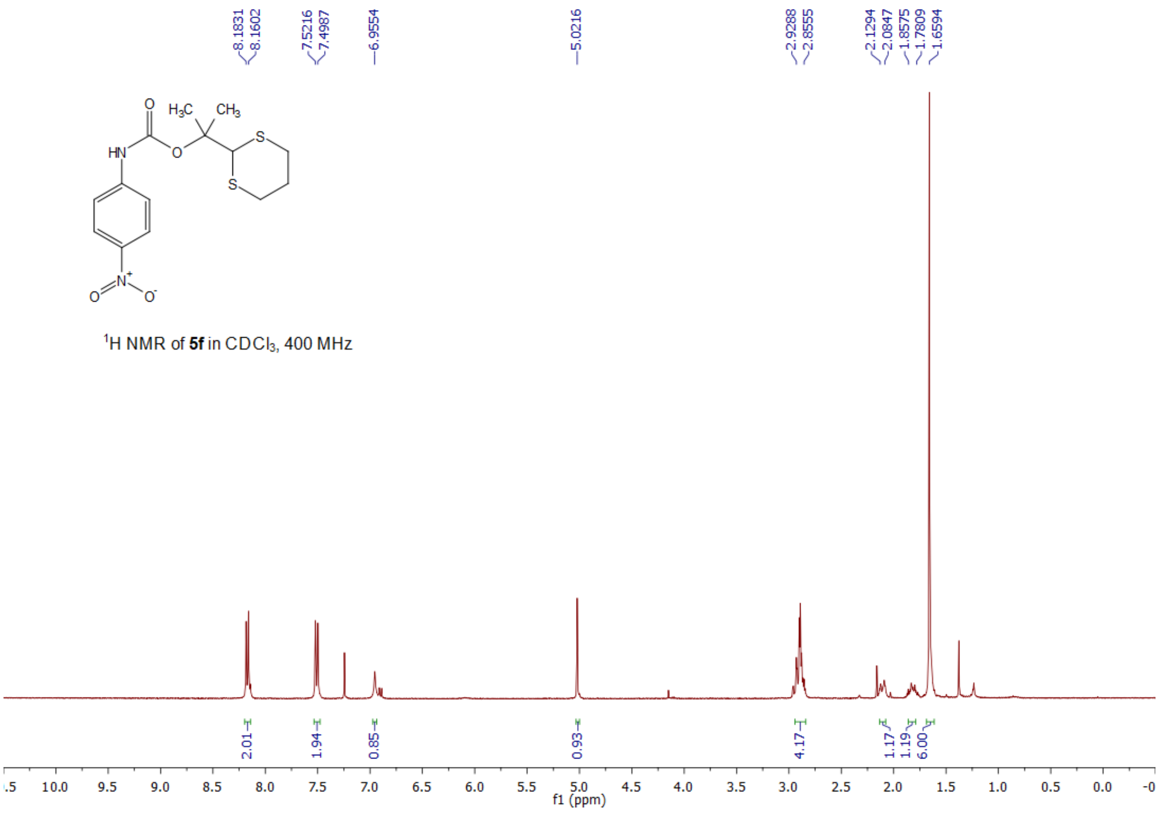

^{1}H NMR of **5f** in $CDCl_3$, 400 MHz

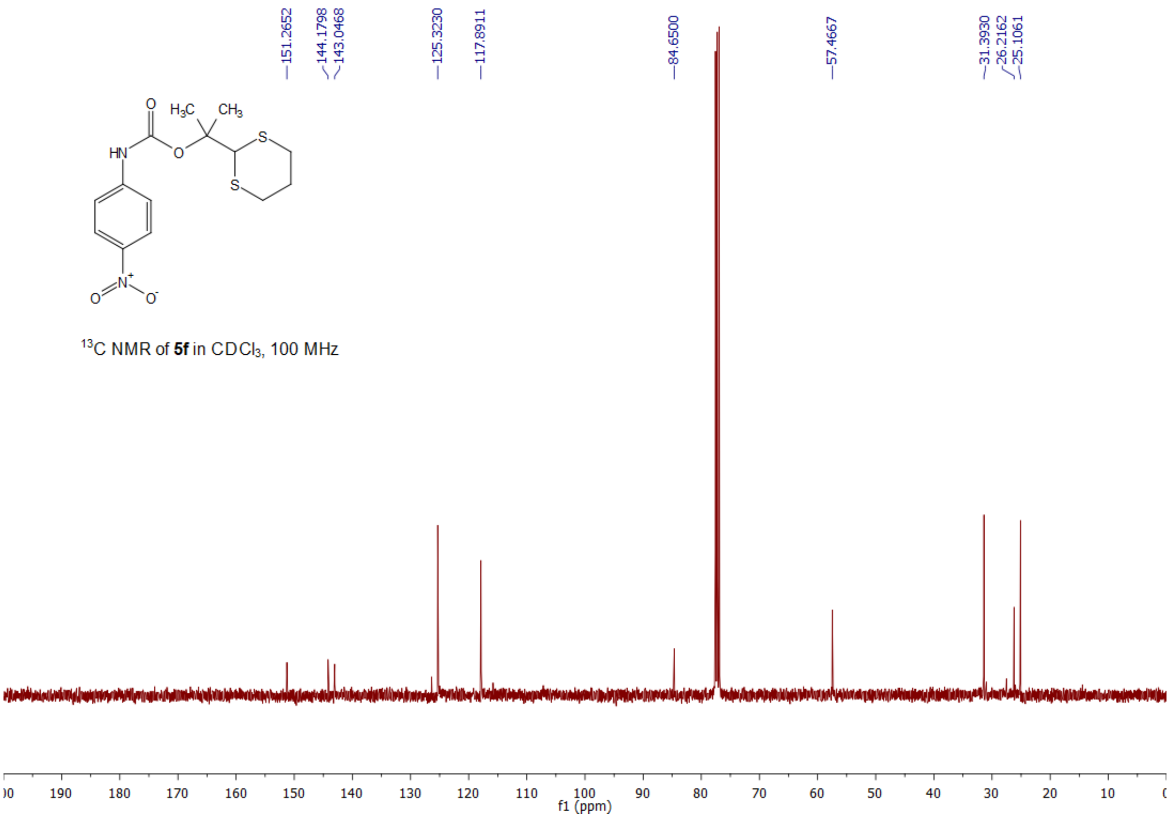

^{13}C NMR of **5f** in $CDCl_3$, 100 MHz

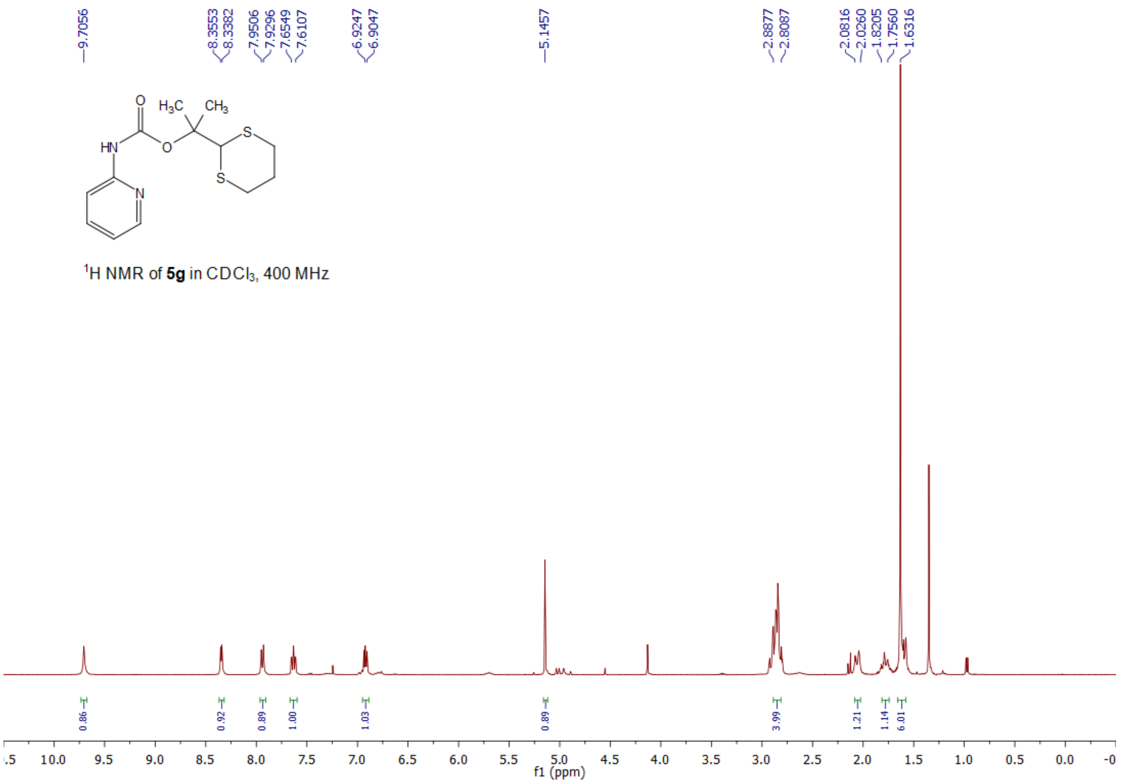

^{1}H NMR of **5g** in $CDCl_3$, 400 MHz

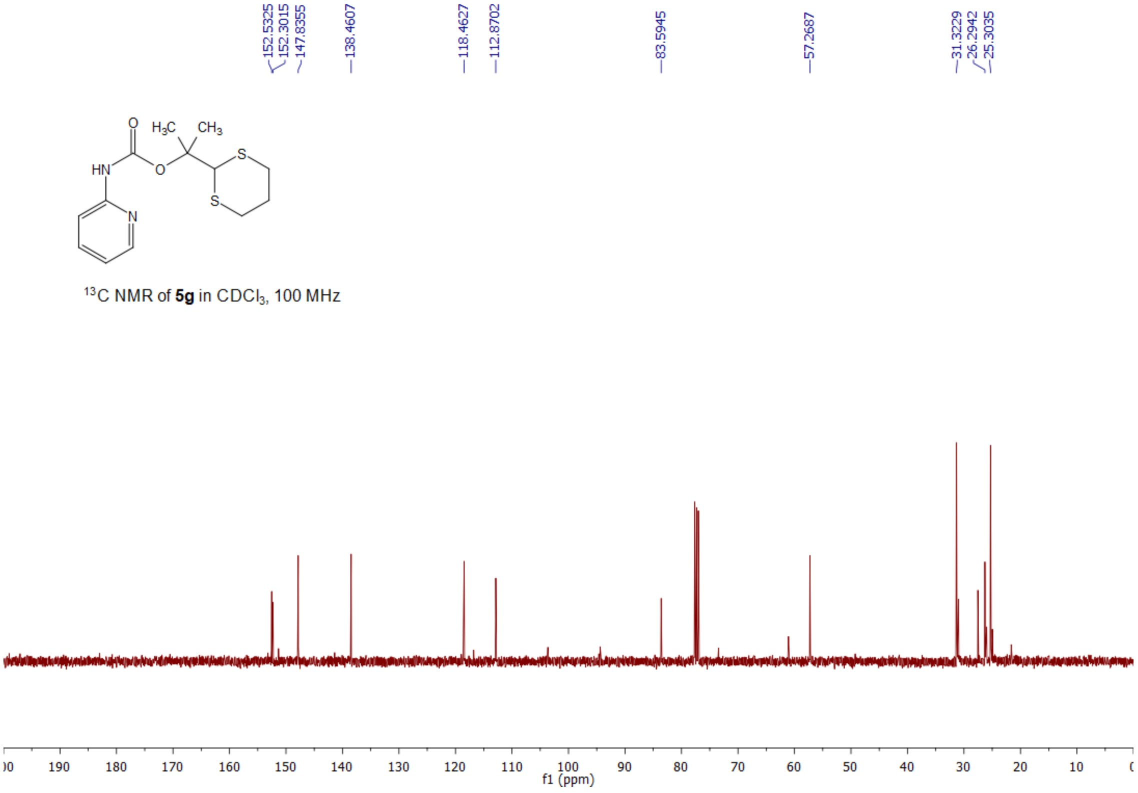

^{13}C NMR of **5g** in $CDCl_3$, 100 MHz

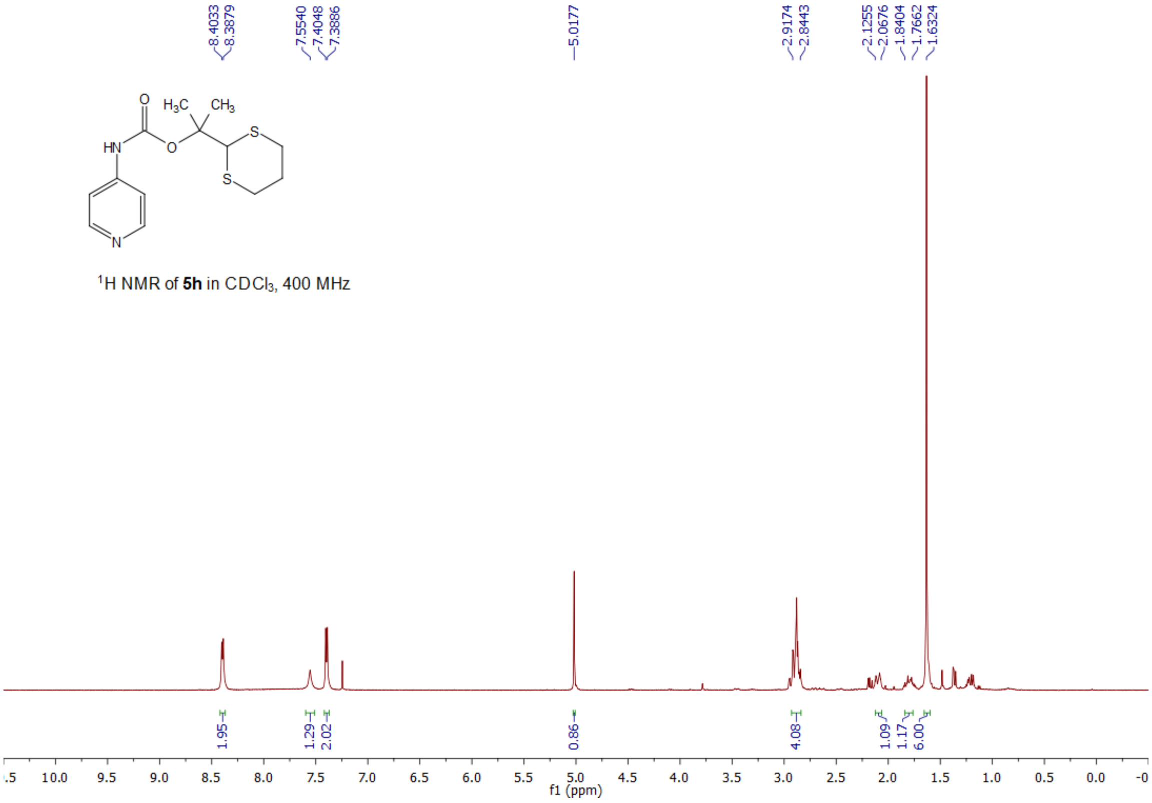

^{1}H NMR of **5h** in $CDCl_3$, 400 MHz

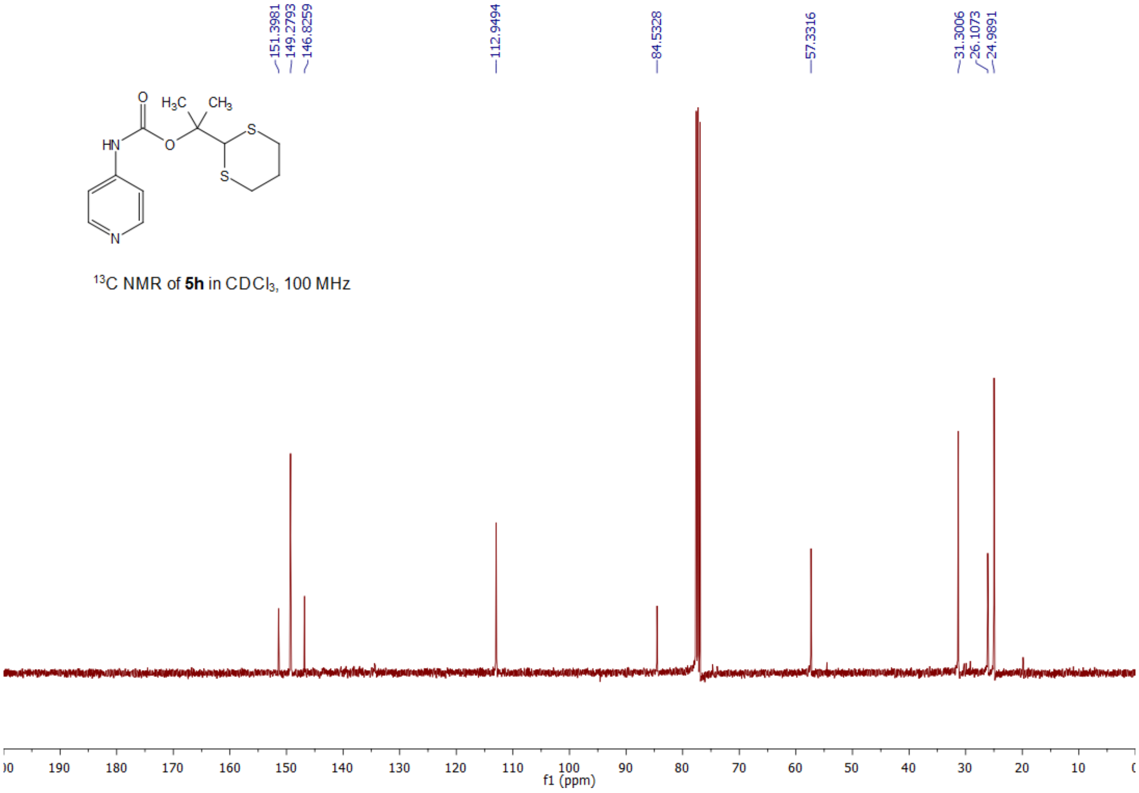

^{13}C NMR of **5h** in $CDCl_3$, 100 MHz

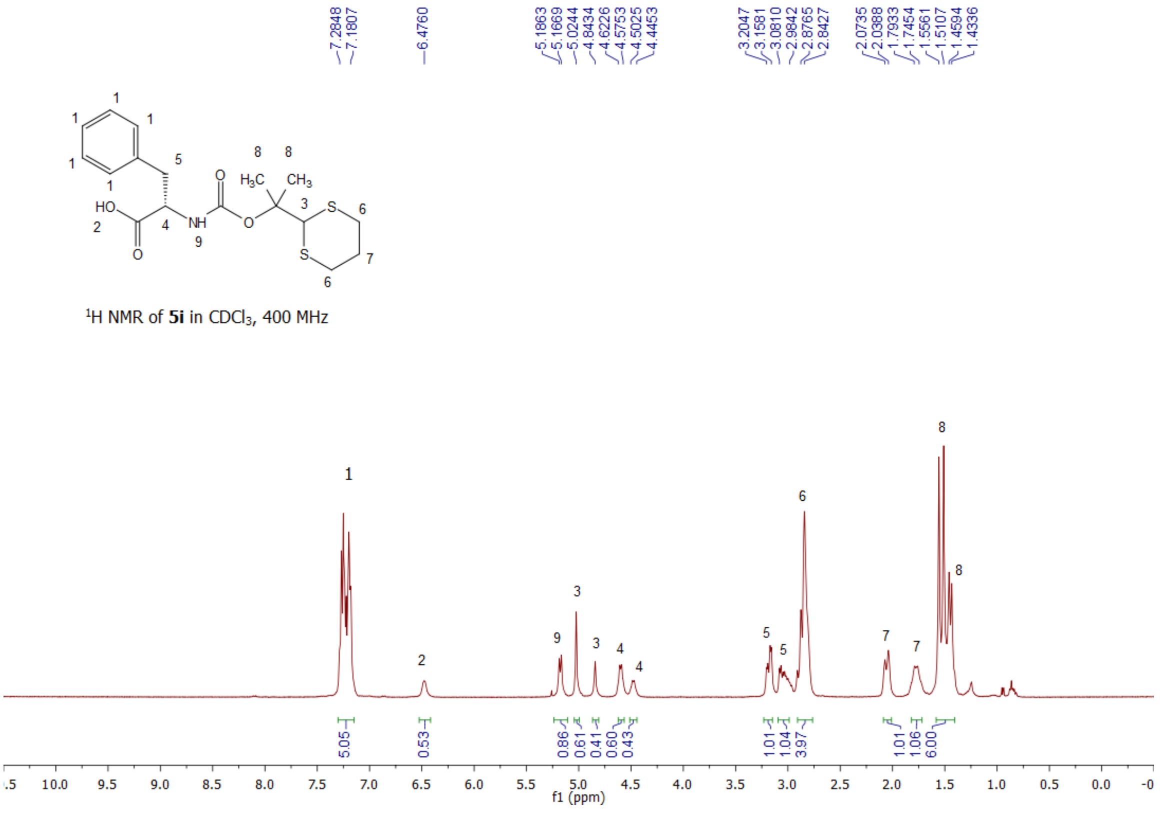

^{1}H NMR of **5i** in $CDCl_3$, 400 MHz

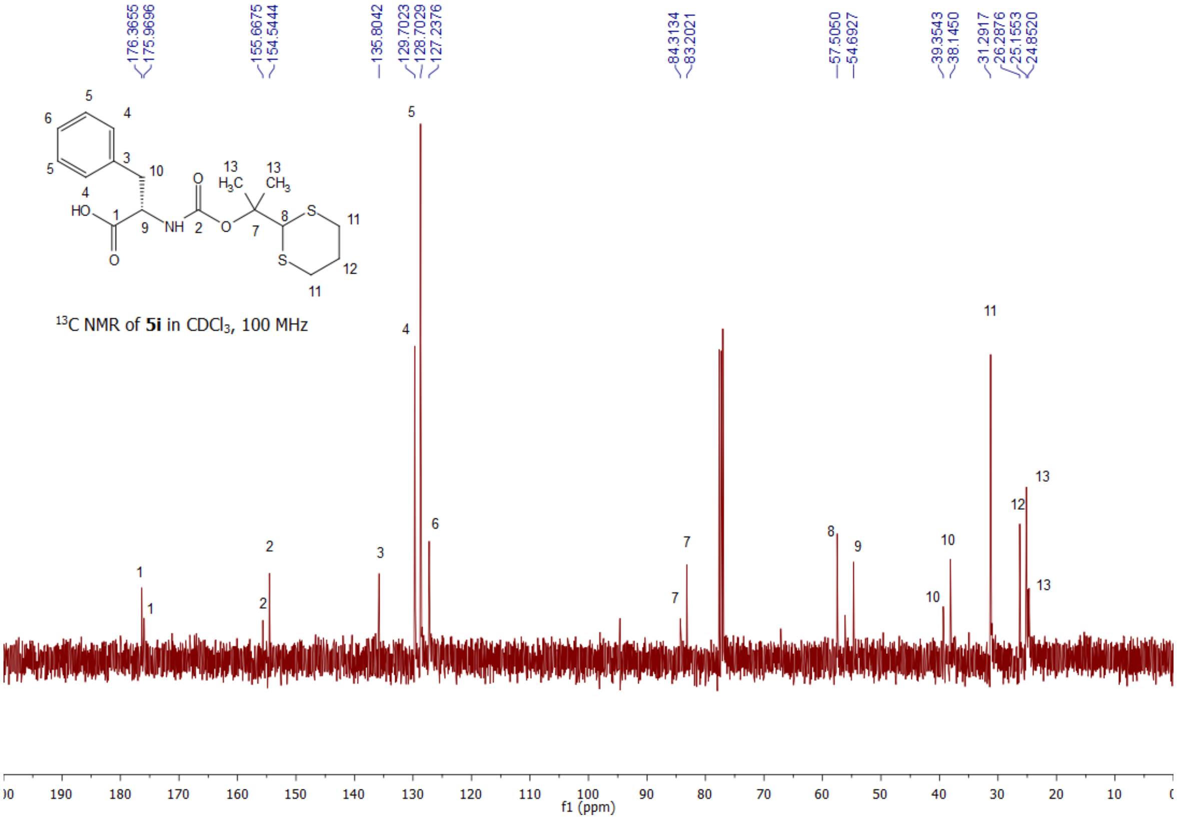

^{13}C NMR of **5i** in $CDCl_3$, 100 MHz

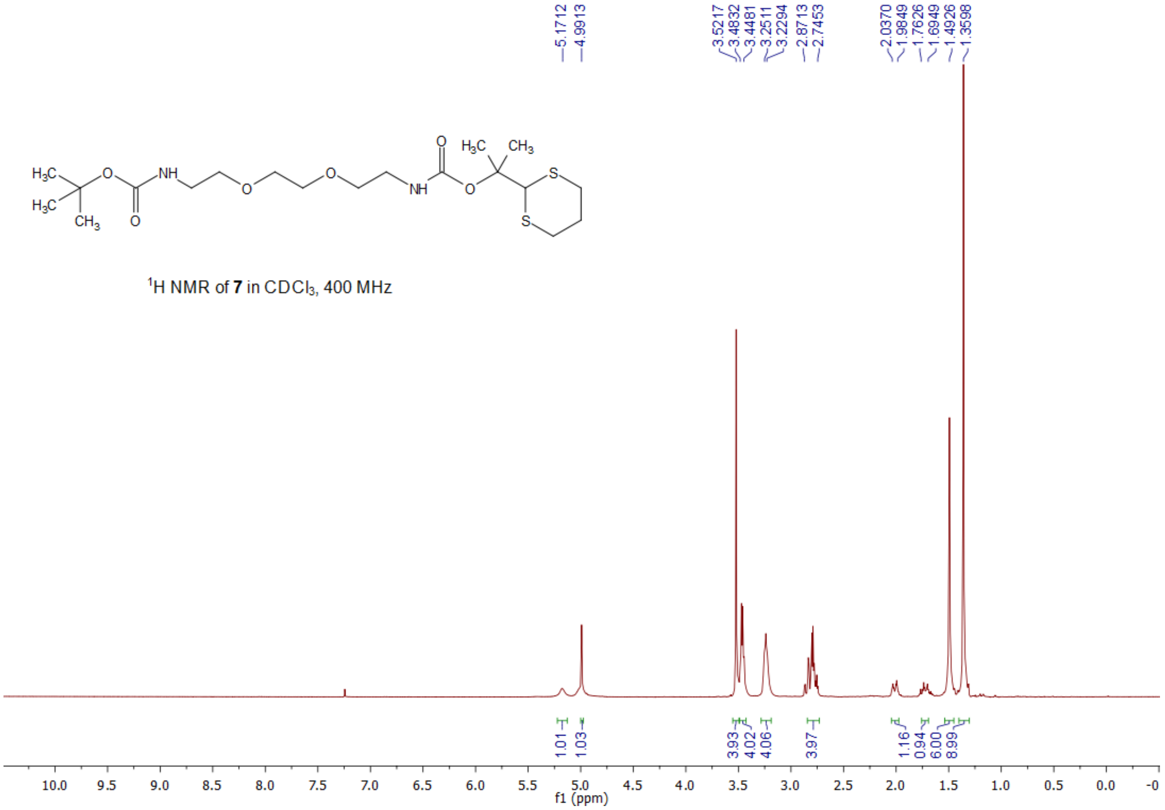

^1H NMR of **7** in $CDCl_3$, 400 MHz

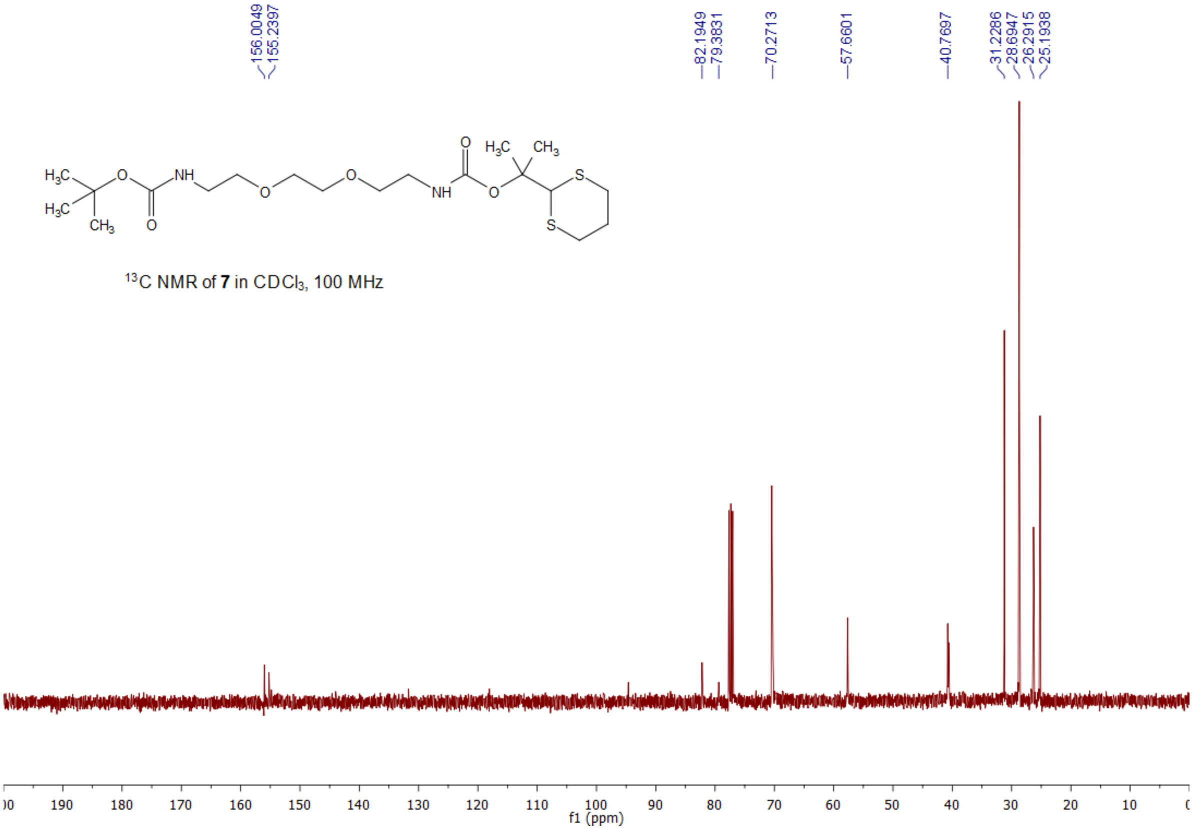

^{13}C NMR of **7** in $CDCl_3$, 100 MHz

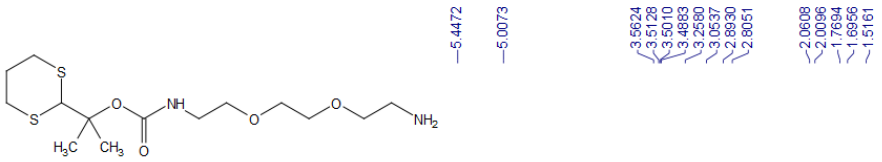

^{1}H NMR of **8** in $CDCl_3$, 400 MHz

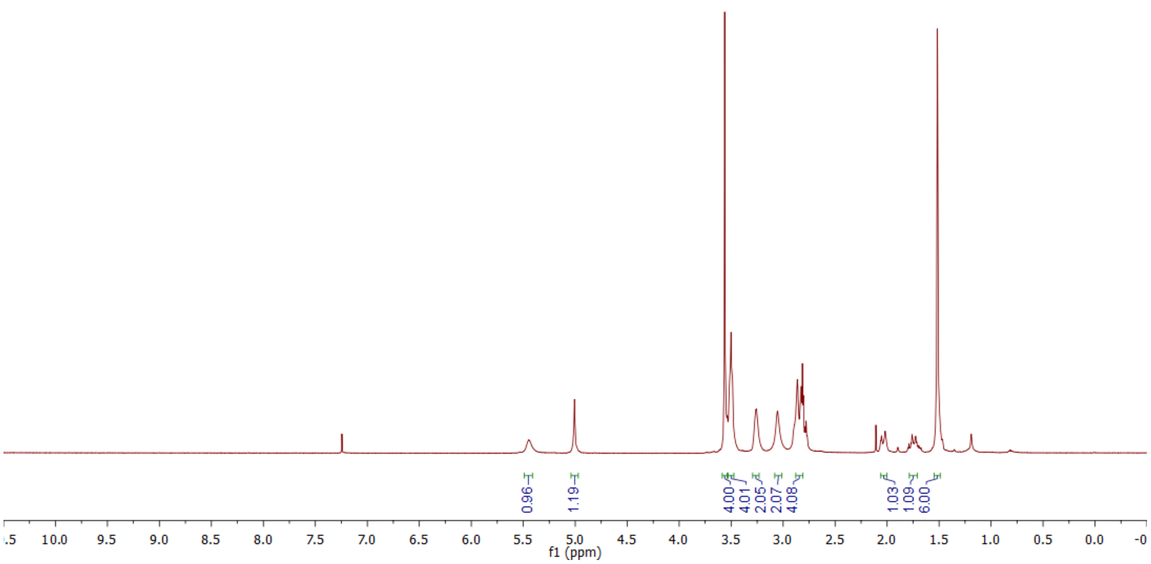

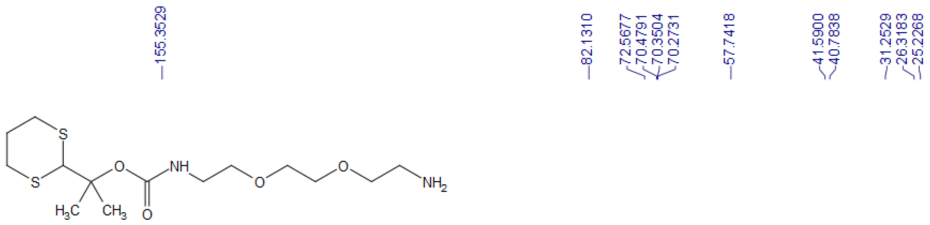

^{13}C NMR of **8** in $CDCl_3$, 100 MHz

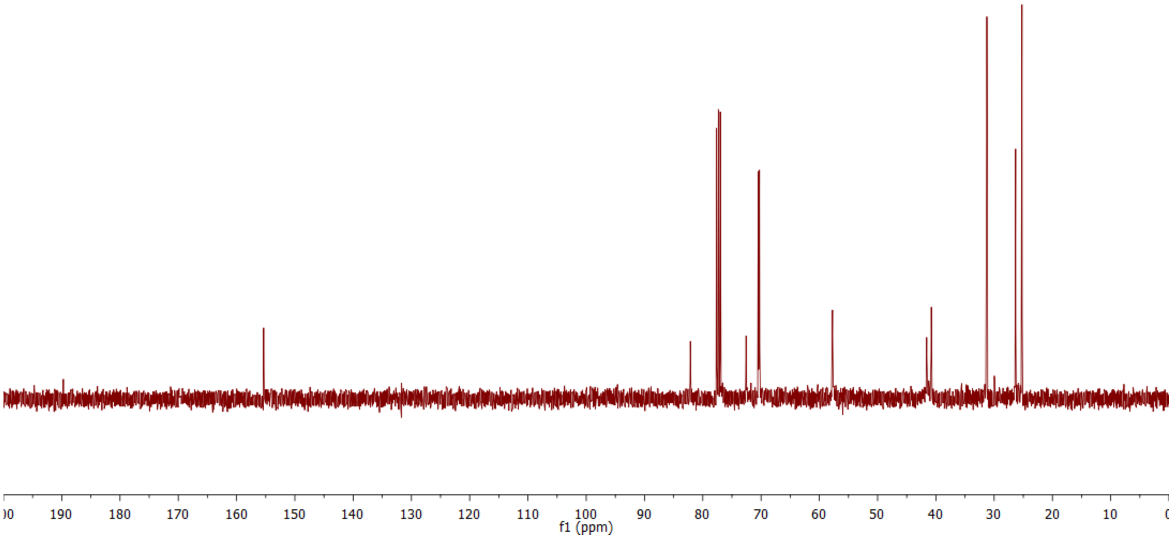

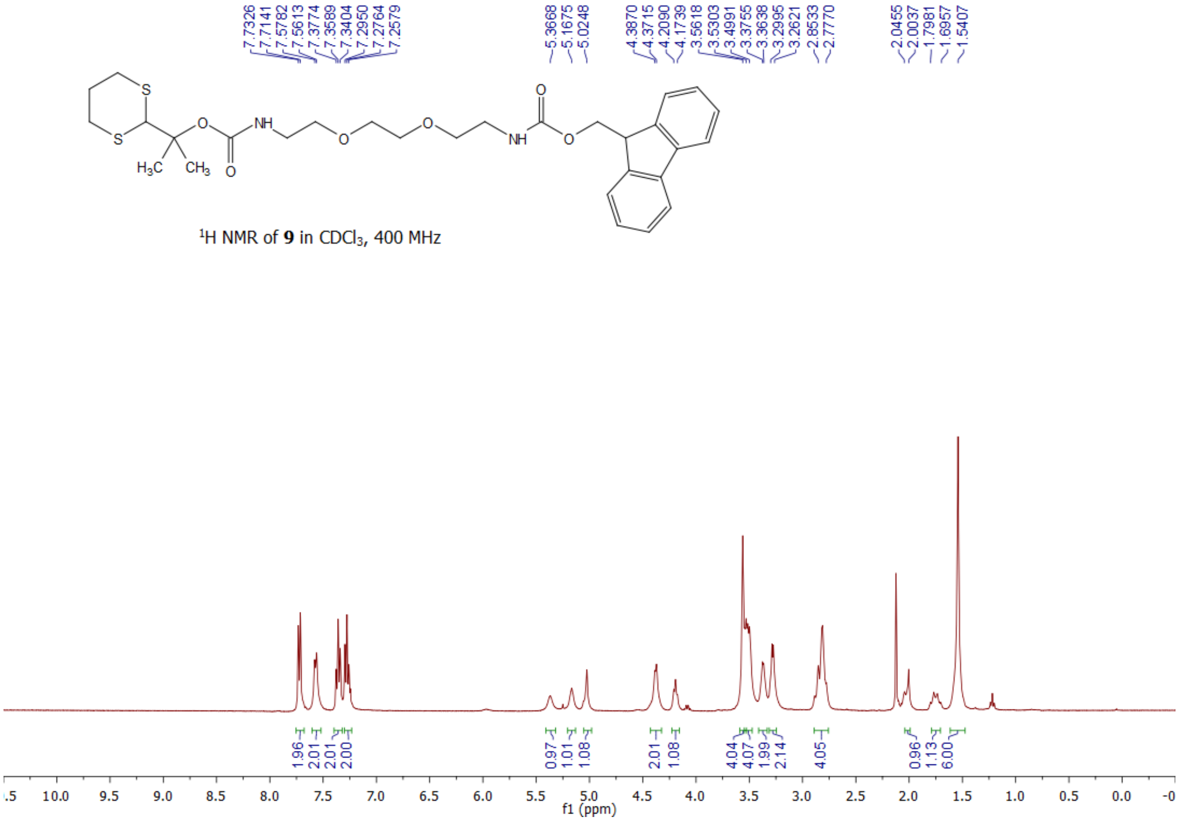

^{1}H NMR of **9** in $CDCl_3$, 400 MHz

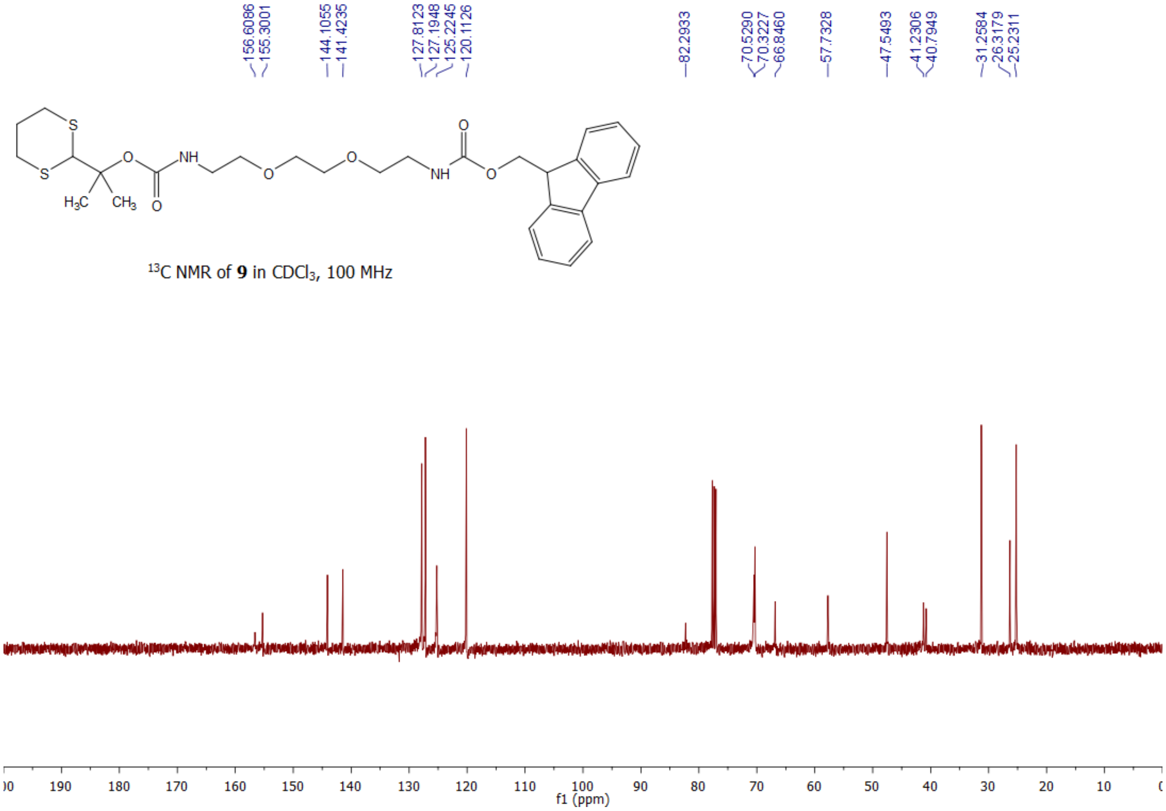

^{13}C NMR of **9** in $CDCl_3$, 100 MHz
